# Supplementary material for: Vacillantins A and B, New Anthrone C-glycosides, and a New Dihydroisocoumarin Glucoside from Aloe vacillans and Its Antioxidant Activities
Source: Plants (Basel). 2020 Nov 24;9(12):1632. doi: 10.3390/plants9121632 (PMC7761211; doi:10.3390/plants9121632)
Supplement: Supplementary file 1 [file plants-09-01632-s001.pdf]

Vacillantins A and B, new anthrone *C*-glycosides, and a new dihydroisocoumarin glucoside from *Aloe vacillans and its antioxidant activities*

Maram Al-Tamimi [1], Shaza M. Al-Massarani [1], Ali A. El-Gamal [1,2,*], Omer A. Basudan [1], Maged S. Abdel-Kader [3,4] and, Wael M. Abdel-Mageed [1,5]

[1] Department of Pharmacognosy, College of Pharmacy, King Saud University, PO. Box 2457, Riyadh, 11451, Saudi Arabia
[2] Department of Pharmacognosy, Mansoura University, Faculty of Pharmacy, El-Mansoura, 35516, Egypt
[3] Pharmacognosy Department, College of Pharmacy, Sattam Bin Abdulaziz University, Al-kharj, 11942, Saudi Arabia
[4] Department of Pharmacognosy, College of Pharmacy, Alexandria University, Alexandria 21215, Egypt
[5] Pharmacognosy Department, Faculty of Pharmacy, Assiut University, Assiut, 71526, Egypt

* Correspondence: aelgamel@ksu.edu.sa; aelgamal00@yahoo.com; Tel.: 00966569780176

Abstract: A new dihydroisocoumarin glucoside, vacillanoside (**3**), and two new anthrone *C*-glycosides microdantin derivatives; vacillantin A (**10**) and B **(11**), together with nine known compounds belonging to the anthraquinone, anthrone and isocoumarin groups were isolated from the leaves of *Aloe vacillans*. The structures were determined based on spectroscopic evidence, including 1D and, 2D nuclear magnetic resonance (NMR) spectroscopy and, high resolution mass spectrometry (HRESIMS) data, along with comparisons to reported data. The leaves were used to extract compounds with different solvents. The extracts tested for antioxidant activity with a variety of *in vitro* tests, including 2,2-diphenyl-1-picrylhydrazyl (DPPH$^{\bullet}$), 2,2′-azino-bis (3-ethylbenzothiazoline-6-sulfonate (ABTS$^{\bullet+}$), ferric reducing antioxidant power assay (FRAP), superoxide and nitric oxide radical scavenging assays. The dichloromethane fraction was most active, displaying significant free radical scavenging activity. The *n*-butanol fraction also showed notable activity in all assays. Therefore, these findings support the potential use of *A. vacillans* leaves as an antioxidant medication due to the presence of polyphenolic compounds.

Keywords: *Aloe vacillans*; Asphodelaceae; dihydroisocoumarin glucoside; anthraquinone;

9 anthrone *C*-glycoside; antioxidant activity.

Content

Antioxidant activity procedures

a- DPPH radical scavenging assay 04
b- ABTS radical cation scavenging assay 04
c- Reducing power assay 04
d- Superoxide radical anion scavenging assay 05
e- Nitric oxide radical scavenging assay 05

Figure 1S. *Aloe vacillans.* 06
Fig. 2S. Chemical structures of the isolated compounds (**1-12**) 07
Table 1S. ^{1}H NMR (CD_3OD, 500 MHz) of compounds (**4-9**) 08
Table 2S.^{13}C NMR (CD_3OD, 125 MHz) of compounds (**4-9**) 09
Figure 3S. ^{1}H NMR spectrum of compound (**3**) (500 MHz, CD_3OD) 10
Figure 4S. ^{13}C NMR spectrum of compound (**3**) (125 MHz, CD_3OD) 10
Figure 5S. DEPT ^{13}C NMR spectrum of compound (**3**) (125 MHz, CD_3OD) 11
Figure 6S. ^{1}H-^{13}C HSQC spectrum of compound (**3**) (500 MHz, CD_3OD) 11
Figure 7S. ^{1}H-^{1}H COSY spectrum of compound (**3**) (500 MHz, CD_3OD) 12
Figure 8S. ^{1}H-^{13}C HMBC spectrum of compound (**3**) (500 MHz, CD_3OD) 12
Figure 9S. HRESIMS spectrum of compound 3 (A) positive mode, (B) negative mode 13
Figure 10S. ^{1}H NMR spectrum of compound (**10**) (500 MHz, CD_3OD) 14
Figure 11S. ^{13}C NMR spectrum of compound (**10**) (125 MHz, CD_3OD) 14
Figure 12S. DEPT ^{13}C NMR spectrum of compound (**10**) (125 MHz, CD_3OD) 15
Figure 13S. ^{1}H-^{13}C HSQC spectrum of compound (**10**) (500 MHz, CD_3OD) 15
Figure 14S. ^{1}H-^{1}H COSY spectrum of compound (**10**) (500 MHz, CD_3OD) 16
Figure 15S. ^{1}H-^{13}C HMBC spectrum of compound (**10**) (500 MHz, CD_3OD) 16
Figure 16S: HRESIMS spectrum of compound (**10**) 17
Figure 17S. ^{1}H NMR spectrum of compound (**11**) (500 MHz, CD_3OD) 18
Figure 18S. ^{13}C NMR spectrum of compound (**11**) (125 MHz, CD_3OD) 18
Figure 19S. DEPT ^{13}C NMR spectrum of compound (**11**) (125 MHz, CD_3OD) 19
Figure 20S. ^{1}H-^{13}C HSQC spectrum of compound (**11**) (500 MHz, CD_3OD) 19
Figure 21S. ^{1}H-^{1}H COSY spectrum of compound (**11**) (500 MHz, CD_3OD) 20
Figure 22S. ^{1}H-^{13}C HMBC spectrum of compound (**11**) (500 MHz, CD_3OD) 21
Figure 23S: HRESIMS spectrum of compound (**11**) 22
Figure 24S: HRESIMS spectrum of the isolated compound 23-25
Figure 25S. Scavenging activity of the organic extracts of *A. vacillans* and ascorbic acid using DPPH assay 26
Figure 26S. Scavenging activity of the organic extracts of *A. vacillans* and ascorbic acid using ABTS assay 27
Figure 27S. Reducing power of the organic extracts of *A. vacillans* and ascorbic acid using FRAP method 28
Figure 28S. Scavenging activity of the different extracts of *A. vacillans* and ascorbic acid using superoxide assay 29
Figure 29S. Scavenging activity of the organic extracts of *A. vacillans* and ascorbic 30

acid using nitric oxide method

a- DPPH(2,2-diphenyl-1-picrylhydrazyl) radical scavenging activity

The antioxidant effect of the plant extracts, based on the scavenging potency of the stable 1, 1-diphenyl-2- picrylhydrazyl (DPPH) free radical, was measured by using the technique approved by Braca *et al.* (2001) [42]. Several concentrations of each extract were mixed with 3 mL of a 0.004% ethanol solution of DPPH. One ml methanol instead of extract was used to prepare control. The absorbance of color strength was measured at 520 nm after 30 minutes and the percentage inhibition of antioxidant effect was measured by using the below formula:

$$[(A0-A1)/ A0] \times 100$$

Where A0 is the absorbance of the control (DPPH solution) and A1 is the absorbance of the oil/standard.

b- ABTS (2,2'-azinobis-(3-ethylbenzothiazoline-6-sulfonic acid) assay

The radical scavenging potency of the Aloe vacillans extracts versus ABTS radical cation was calculated using the technique designated by Re *et al.* (1999) [43]. The ABTS solution was prepared in water with a concentration of 7 mmol/L; an aqueous solution of potassium persulphate was also prepared with a concentration of 2.45 mmol/L. The two solutions were added in equal volume (1: 1) and stored in dark for 6 hr. at room temperature. During that period, ABTS radical was produced. The ABTS stock solution was diluted with ethanol to an absorbance of 0.70±0.02 at 734 nm and equilibrated at 30°C. An aliquot of different extracts was mixed with 2.9 ml of diluted ABTS radical cation solution. After the reaction was incubated at 30°C for 20 minutes, absorbance was measured at 734 nm. The ability affinity of the oil to quench ABTS free radical was calculated according to the formula:

$$\text{Scavenging (\%)} = [(Ac- Aa/ Ac)] \times 100$$

Where Ac= absorbance of control and Aa= absorbance of the oil.

c- Ferric reducing antioxidant power (FRAP) assay

The ferric free radical scavenging power was measured based on the technique described by Oyaizu (1986) [44]. The reduction of ferric ion to ferrous ion is confirmed by formation of Perl's Prussian blue color. several dilution of the *Aloe vacillans* extracts (20-100 µg/ml) in 1 mL of distilled water were added to 0.2 M phosphate buffer (2.5 mL, pH 6.6) and 1 % potassium ferricyanide (2.5 mL). The mixture was incubated at 50°C for 20 minutes. 2.5 ml of 10 % trichloroacetic acid was added to the mixture, followed by centrifugation at 3000 rpm for 10 minutes. 2.5 ml of distilled water was added to equal amount of the supernatant followed by addition of 0.5 ml of 0.1 % $FeCl_3$, the absorbance was recorded at 700 nm.

d- Superoxide Anion Scavenging Radical Assay

Reactive oxygen species such as superoxide anions and other free radicals are formed during metabolism and specialized physiological reactions. Repeated exposure to these radicals is considered a main cause of aging, neurodegenerative, and inflammatory diseases due to gradual damage of cellular components, such as DNA and proteins [45].

The superoxide anion radical scavenging activities of the extracts were evaluated using the method described by Fontana et al [45] with slight modification. To various concentrations of the samples (20.0–100 _µg/mL), 1.0 mL of phosphate buffer (0.1 M, pH 7.2), 1.0 mL of NADH (2 mM), 1.0 mL of NBT (0.5 mM), and 0.1 mL of PMS (0.03 mM) were added. After 5 min incubation at ambient temperature, the absorbance was read at 562 nm against a reagent blank to detect the quantity of formazan generated. The standard used was ascorbic acid. All of the tests were performed in triplicate.

The % scavenging/inhibition were calculated as below

% scavenging/inhibition = [(Absorbance Control- Absorbance Test)/Absorbance control] _ 100 (2)

where A control = absorbance of control sample and Atest = absorbance in the presence of extracts or standard.

e- Nitric Oxide Scavenging Radical Assay

Nitric oxide is classified as a free radical because of its unpaired electron and important reactivity with certain types of proteins and other free radicals, such as superoxide in vivo. NO is synthesized in the vascular endothelial cells, certain neuronal cells, and phagocytes. Chronic exposure to nitric oxide radical can cause various carcinomas and inflammatory conditions [46]. In vitro quenching of NO radical is one of the methods that can be used to measure antioxidant activity in which nitric oxide is generated from sodium nitroprusside interaction with oxygen to produce nitrite ions, which were measured by the Griess reaction. The procedure done was reported by Nagmoti et al with slight modifications [46]. Three milliliters of 10 mM sodium nitroprusside in phosphate buffered saline (pH 7.4) were added to different concentrations of (20–100 µg/mL) tested samples. After 60 min incubation at 25 _C, the resulting solution was then added to 5.0 mL of Griess reagent (1% sulphanilamide, 0.1% NEDD in 2% H_3PO4). At 546 nm, the absorbance of the chromophore formed was measured against a reagent blank. Percentage inhibition of the nitrite ions generated was observed. Ascorbic acid was used as a standard for comparison. The free radical
scavenging activity was determined by computing % inhibition as above.

Refrences

42. Braca, A.; Tommasi, N.D.; Bari, L.D.; Pizza, C.; Politi, M.; Morelli, I. Antioxidant principles from *Bauhinia terapotensis*. *J. Nat. Prod.* **2001**, 64, 892–895.

43. Re, R.; Pellegrini, N.; Proteggente, A.; Pannala, A.; Yang, M.; Rice-Evans, C. Antioxidant activity

applying an improved ABTS radical cation decolorization assay. *Free Radic. Biol. Med.* **1999**, *26*, 1231–1237.

44. Oyaizu, M. Studies on products of browning reactions. Antioxidative activities of products of browning reaction prepared from glucosamine. *Jpn. J. Nutr.* **1986**, *44*, 307–315.
45. Fontana, L.; Giagulli, C.; Minuz, P.; Lechi, A.; Laudanna, C. 8-Iso-PGF2 alpha induces beta 2-integrinmediated rapid adhesion of human polymorphonuclear neutrophils: A link between oxidative stress and ischemia/reperfusion injury. *Arterioschler Thromb. Vasc. Biol.* **2001**, *21*, 55–60.
46. Nagmoti, D.M.; Khatri, D.K.; Juvekar, P.R.; Juvekar, A.R. Antioxidant activity and free radical scavenging potential of *Pithecellobium dulce* Benth seed extracts. *Free Rad. Antiox*. **2011**, 2, 37–43.

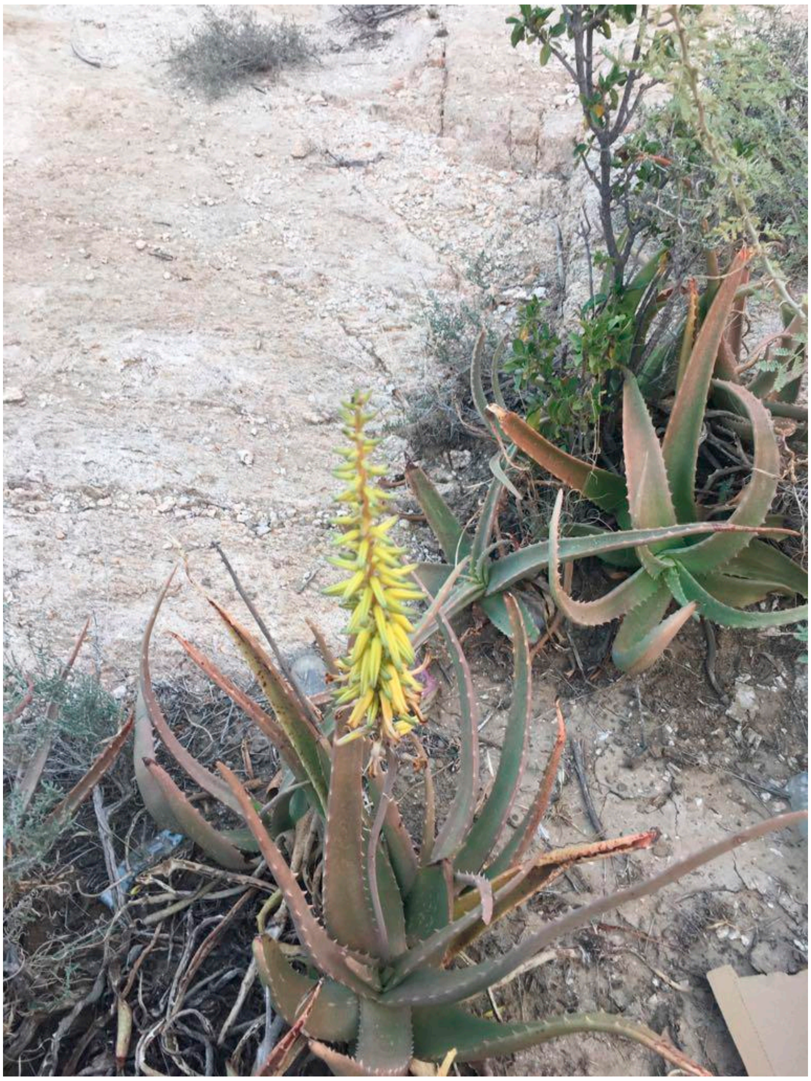

Figure 1S. *Aloe vacillans*.

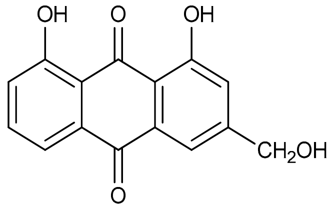

1

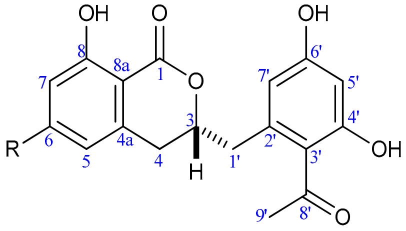

2 R = OH
3 R = Glc

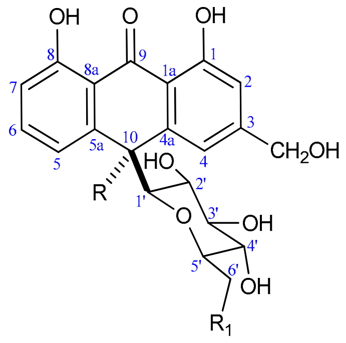

4 R = OH, R_1 = OH
6 R = H, R_1 = OH
8 R = H, R_1 = coumaroyl
10 R = H, R_1 = caffeoyl

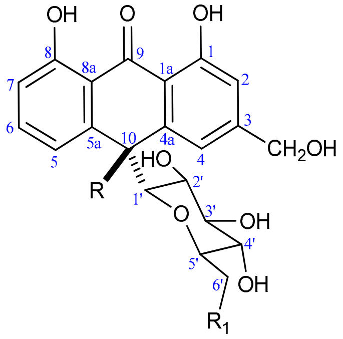

5 R = OH, R_1 = OH
7 R = H, R_1 = OH
9 R = H, R_1 = coumaroyl
11 R = H, R_1 = caffeoyl

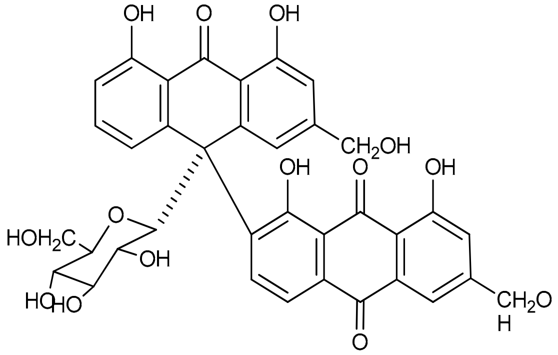

12

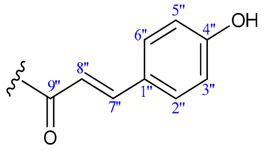

coumaroyl

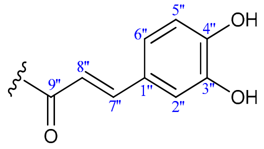

caffeoyl

Fig. 2S. Chemical structures of the isolated compounds (**1-12**) from *A. vacillans* (Glc = glucose).

Table 1S. ^{1}H NMR (CD_3OD, 500 MHz) of compounds (**4-9**).

No.	4	5	6	7	8	9
**1**	-	-	-	-	-	-
**2**	6.94, br s	6.94, br s	6.87, br s	6.86, br s	6.81, br s	6.75, br s
**3**	-	-	-	-	-	-
**4**	7.38, br s	7.49, br s	7.02, br s	7.03, br s	6.98, br s	6.97, br s
**5**	7.46, d (7.6)	7.39, d (7.6)	7.01, br d (7.0)	7.06, d (7.4)	6.91, br d (7.4)	7.00, d (7.3)
**6**	7.55, t (8.1, 7.9)	7.57, t (8.0)	7.45, t (8.0)	7.44, t (8.1)	7.40, t (7.8)	7.37*
**7**	6.91, d (8.2)	6.93, d (7.9)	6.83, d (8.3)	6.84, d (8.1)	6.70, d (8.2)	6.77, d (8.5)
**8**	-	-	-	-	-	-
**9**	-	-	-	-	-	-
**10**	-	-	4.55, br s	4.56, (br s)	4.46, d (1.6)	4.50, br s
**1a**	-	-	-	-	-	-
**4a**	-	-	-	-	-	-
**5a**	-	-	-	-	-	-
**8a**	-	-	-	-	-	-
**3-CH₂OH**	4.66, d (14.6)<br>4.70, d (14.6)	4.64, d (14.6)<br>4.68, d (14.4)	4.65, d (5.0)	4.64, d (2.1)	4.62, d (5.9)	4.64, d (2.1)
**H-1`**	3.27, d (9.5)	3.26, d (9.3)	3.30, dd (9.3, 1.9)	3.39, dd (9.8, 2.0)	3.31, d (9.5)	3.31, dd (9.8, 2.0)
**H-2`**	2.94, br t (9.1)	2.97, t (9.2)	2.91, t (9.3)	3.00, t (9.3)	3.00, t (9.3)	3.09, t (9.3)
**H-3`**	3.24, t (8.9)	3.24, d (8.4)	3.28, t (8.7)	3.26, m	3.29, d (9.3)	3.31, m
**H-4`**	2.82, t (9.4)	2.82, dd (9.4, 9.0)	2.83, t (9.2)	2.90, br d (5.5)	2.86, t (9.2)	2.67, t (9.2)
**H-5`**	2.94, d (9.1)	2.92, m	2.82, m	2.90, br d (5.5)	3.02, dd (9.3, 2.5)	3.04, t (8.0)
**H-6`a**	3.57, dd (11.7, 2.0)	3.55, dd (11.7, 2.1)	3.47, br dd (12.1, 1.3)	3.36, dd (11.7, 4.9)	3.82, dd (11.7, 7.0)	3.82, dd (11.4, 7.0)
**H-6`b**	3.35, m	3.35, dd (11.8, 6.2)	3.32, br dd (9.8, 2.3)	3.54, br d (11.0)	4.23, dd (11.7, 2.0)	4.23, br d (11.5)
**Acyl moiety**					Coumaroyl	Coumaroyl
**1``**	-	-	-	-	-	-
**2``**	-	-	-	-	7.49, d (8.5)	7.48, d (8.4)
**3``**	-	-	-	-	6.85, d (8.5)	6.68, d (8.4)
**4``**	-	-	-	-	-	-
**5``**	-	-	-	-	6.85, d (8.5)	6.68, d (8.4)
**6``**	-	-	-	-	7.49, d (8.5)	7.48, d (8.4)
**7``**	-	-	-	-	7.40, d (15.9)	7.40, d (15.6)
**8``**	-	-	-	-	6.13, d (15.9)	6.12, d (5.9)
**9``**	-	-	-	-	-	-

Table 2S. ^{13}C NMR (CD_3OD, 125 MHz) of compounds (**4-9**).

**No.**	**4**	**5**	**6**	**7**	**8**	**9**
1- C	163.1	162.9	163.2	163.0	163.2	162.8
2- CH	115.3	115.2	114.4	114.1	114.6	114.1
3- C	151.6	152.4	151.4	152.3	151.0	152.1
4- CH	116.9	116.0	119.2	117.7	119.2	119.1
5- CH	118.1	119.0	120.0	121.3	119.4	121.3
6- CH	137.1	136.5	137.0	136.3	136.9	136.0
7- CH	117.9	118.2	116.8	117.1	116.7	117.2
8- C	162.5	162.8	162.8	163.0	162.6	162.7
9- C	194.4	194.4	195.4	195.4	195.3	195.3
10- CH	76.6	76.8	45.8	45.7	45.5	45.4
1a- C	116.4	116.1	117.6	117.5	117.9	117.8
4a- C	146.8	146.6	143.1	142.9	142.4	142.1
5a- C	148.6	149.0	146.5	146.8	146.9	147.2
8a- C	117.1	117.6	118.5	118.8	118.9	119.1
11- CH_2	64.6	64.6	64.5	64.5	64.5	64.5
1\`- CH	85.2	85.1	86.5	86.5	85.8	85.8
2\`- CH	72.9	72.9	71.8	71.8	715	71.5
3\`- CH	79.4	79.5	79.9	79.9	79.7	79.7
4\`- CH	71.5	71.5	71.9	71.9	71.8	71.8
5\`- CH	81.6	81.6	81.6	81.5	79.0	78.9
6\`- CH_2	63.2	63.2	63.2	63.1	64.5	64.5
**Acyl moiety**					Coumaroyl	Coumaroyl
1\`\`- C	-	-	-	-	127.3	127.3
2\`\`- CH	-	-	-	-	131.3	131.4
3\`\`- C	-	-	-	-	116.7	116.8
4\`\`- C	-	-	-	-	161.1	161.0
5\`\`- CH	-	-	-	-	116.7	116.8
6\`\`- CH	-	-	-	-	131.3	131.4
7\`\`- CH	-	-	-	-	146.4	146.5
8\`\`- CH	-	-	-	-	114.9	114.8
9\`\`- C	-	-	-	-	168.9	169.0

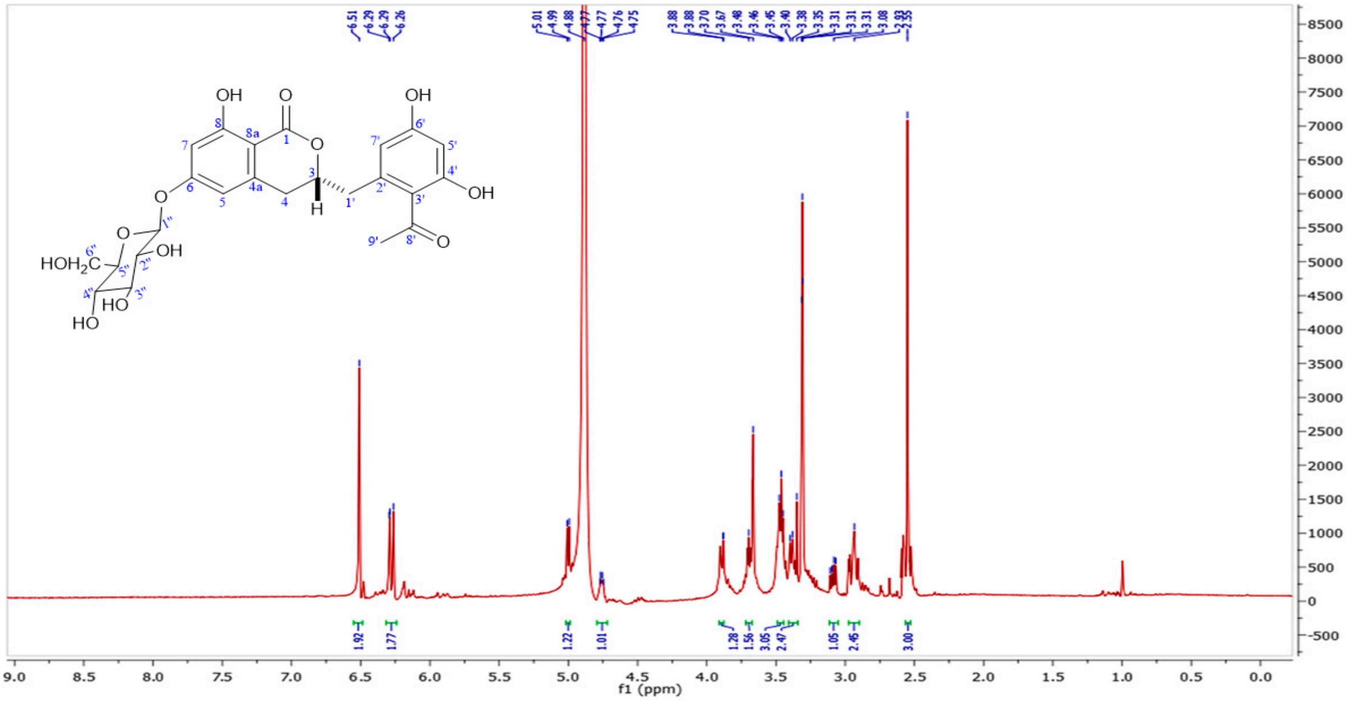

Figure 3S. ^{1}H NMR spectrum of compound (**3**) (500 MHz, CD_3OD)

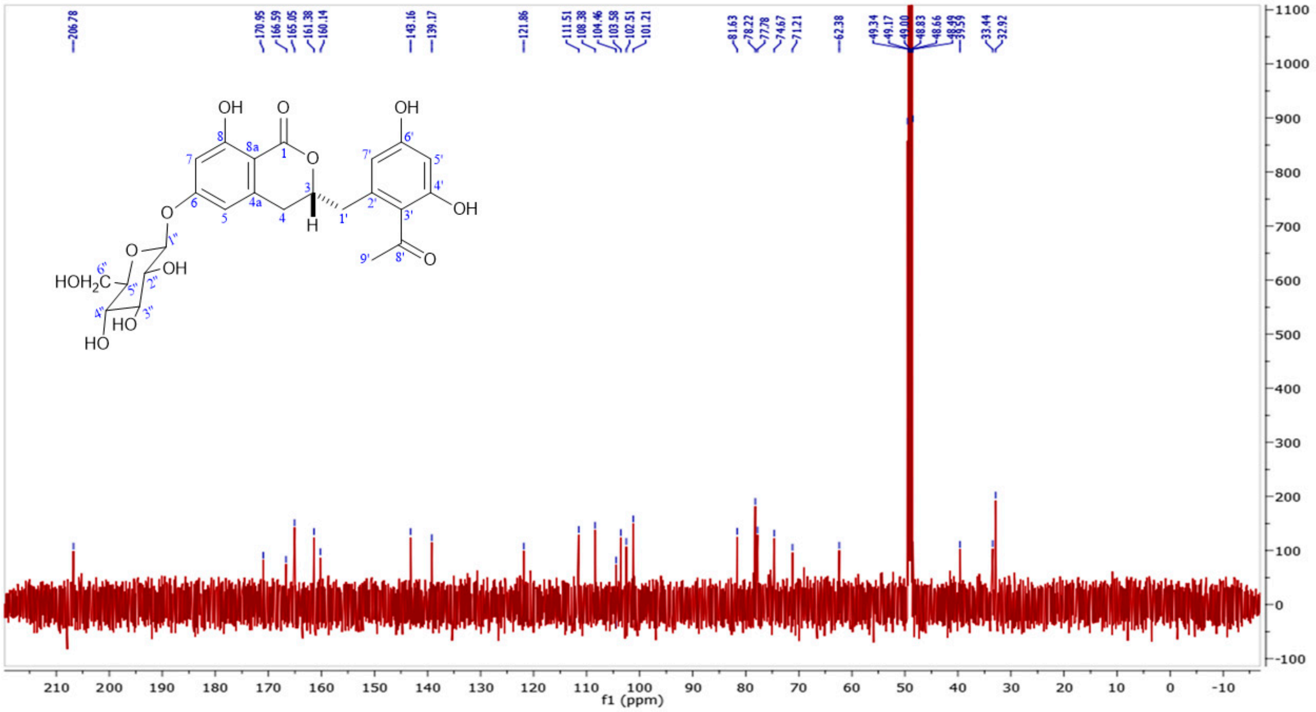

Figure 4S. ^{13}C NMR spectrum of compound (**3**) (125 MHz, CD_3OD)

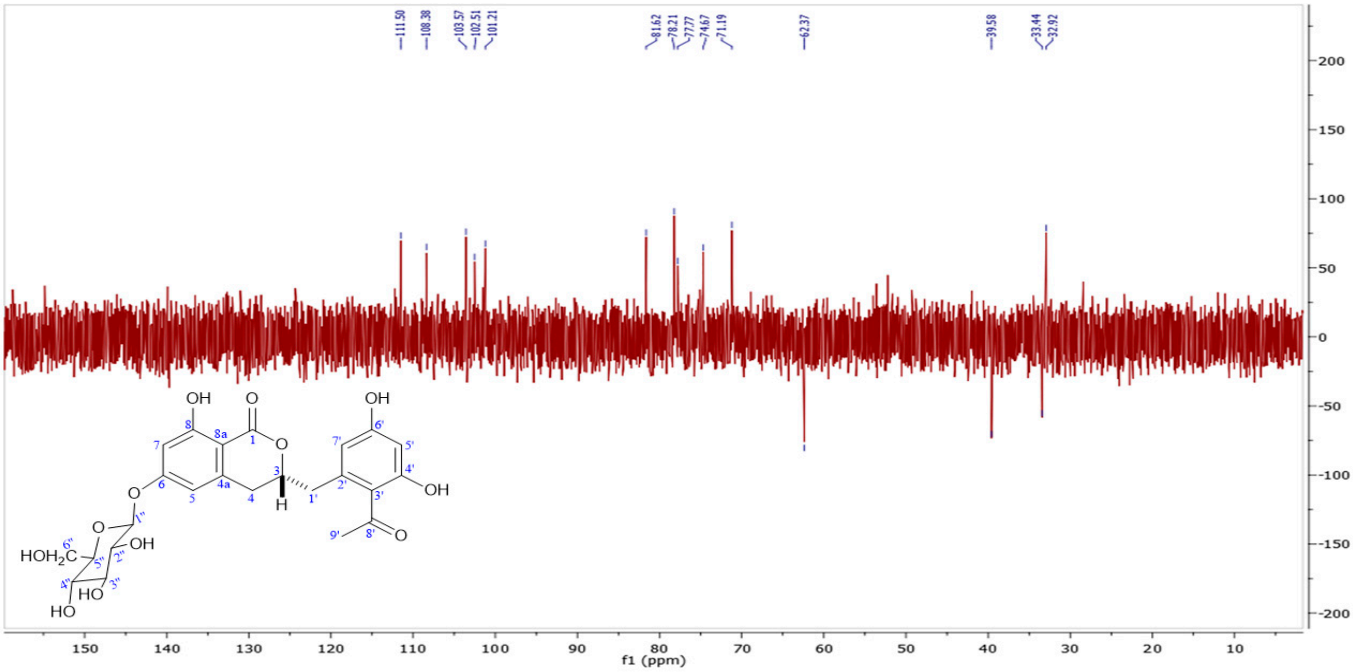

Figure 5S. DEPT ^{13}C NMR spectrum of compound (**3**) (125 MHz, CD_3OD)

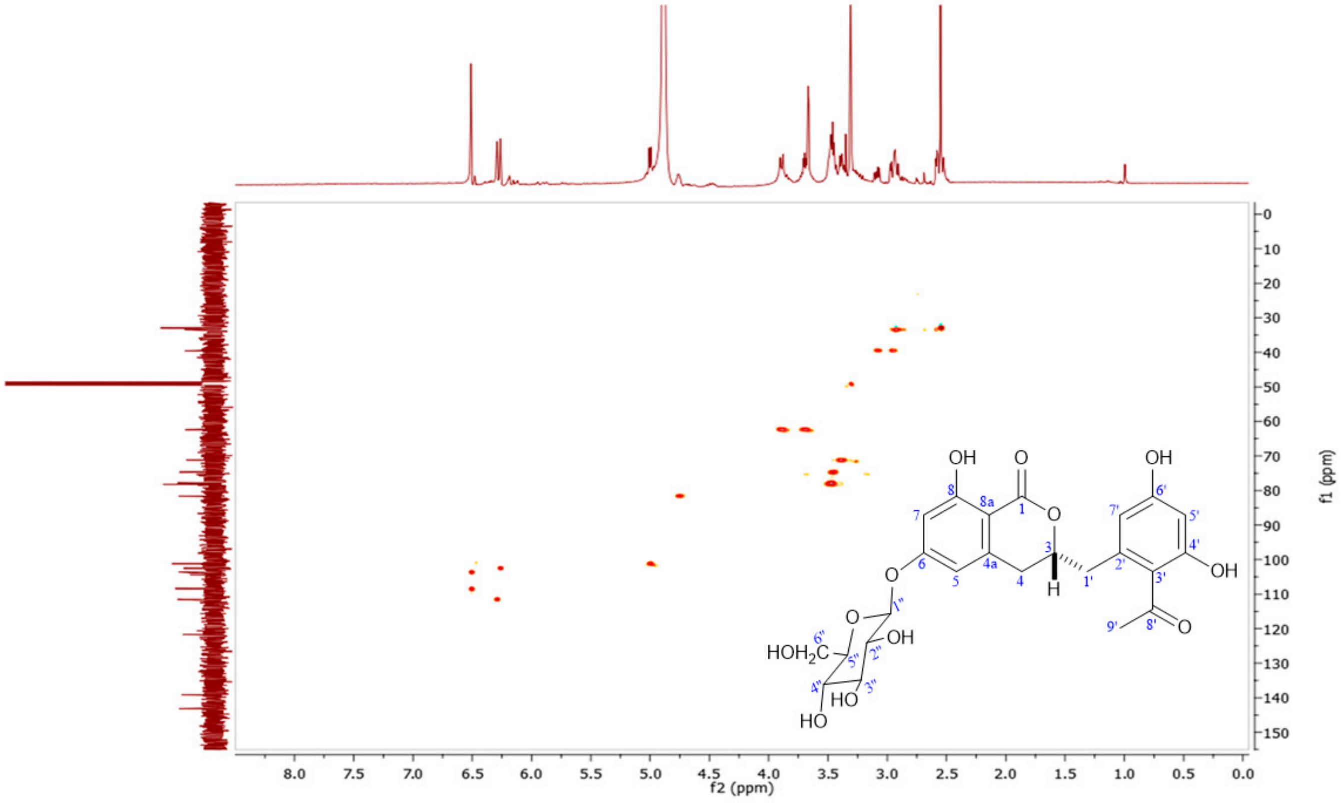

Figure 6S. ^{1}H-^{13}C HSQC spectrum of compound (**3**) (500 MHz, CD_3OD)

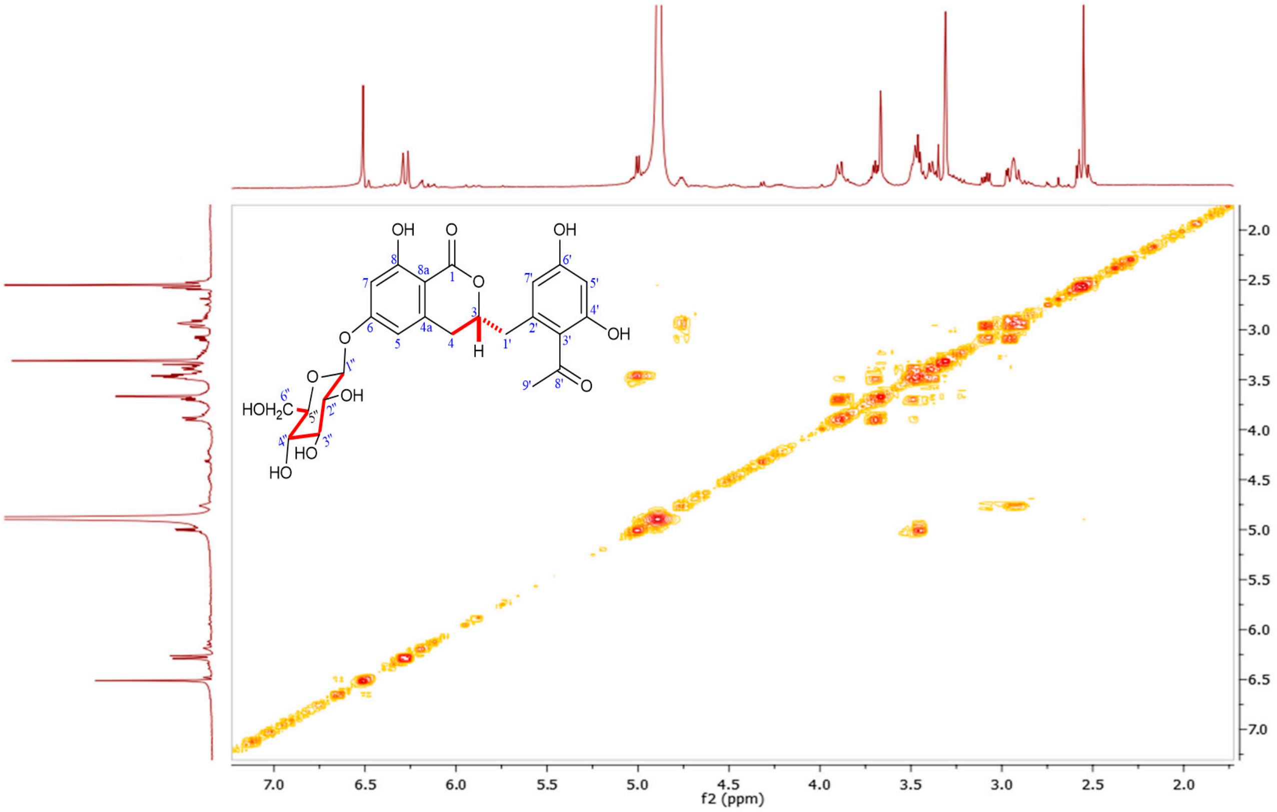

Figure 7S. ^{1}H-^{1}H COSY spectrum of compound (**3**) (500 MHz, CD3OD)

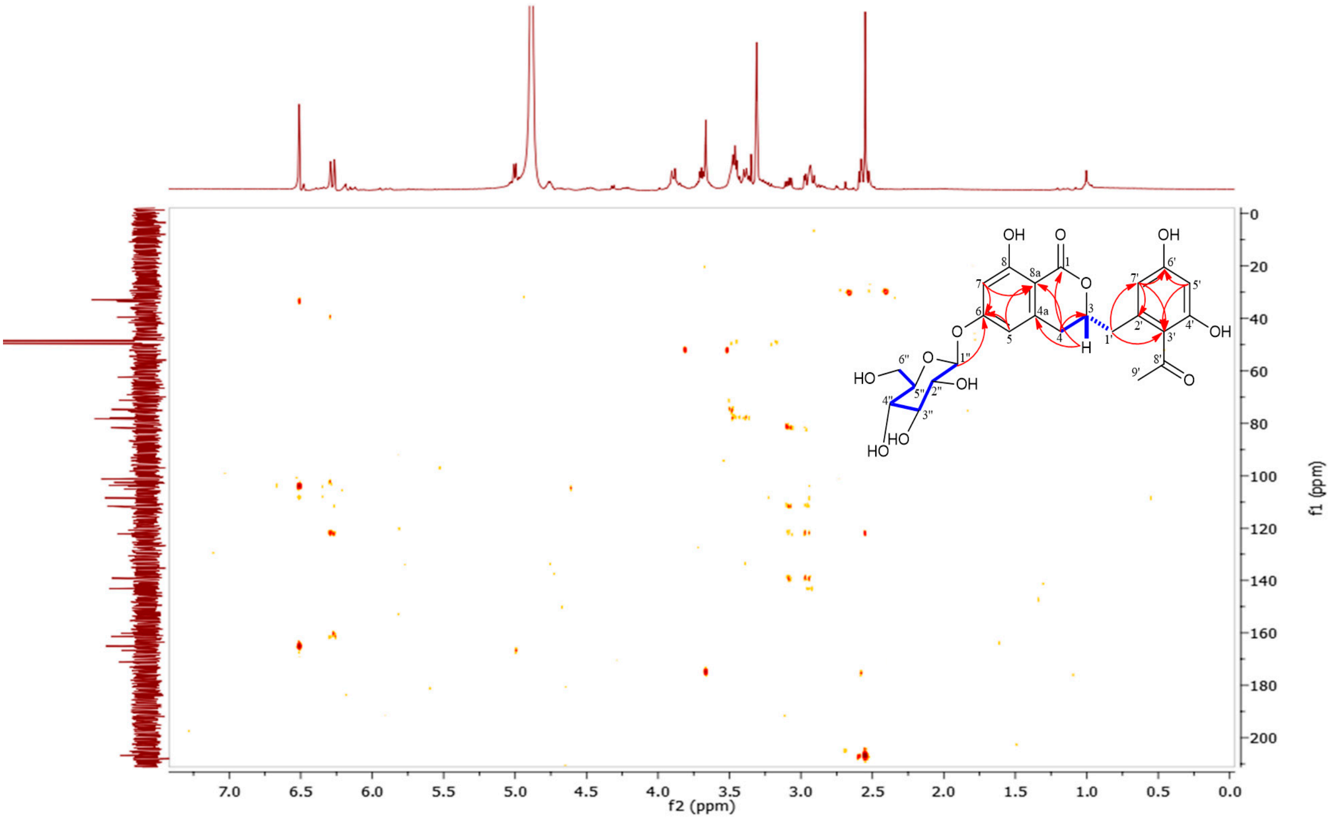

Figure 8S. ^{1}H-^{13}C HMBC spectrum of compound (**3**) (500 MHz, CD3OD)

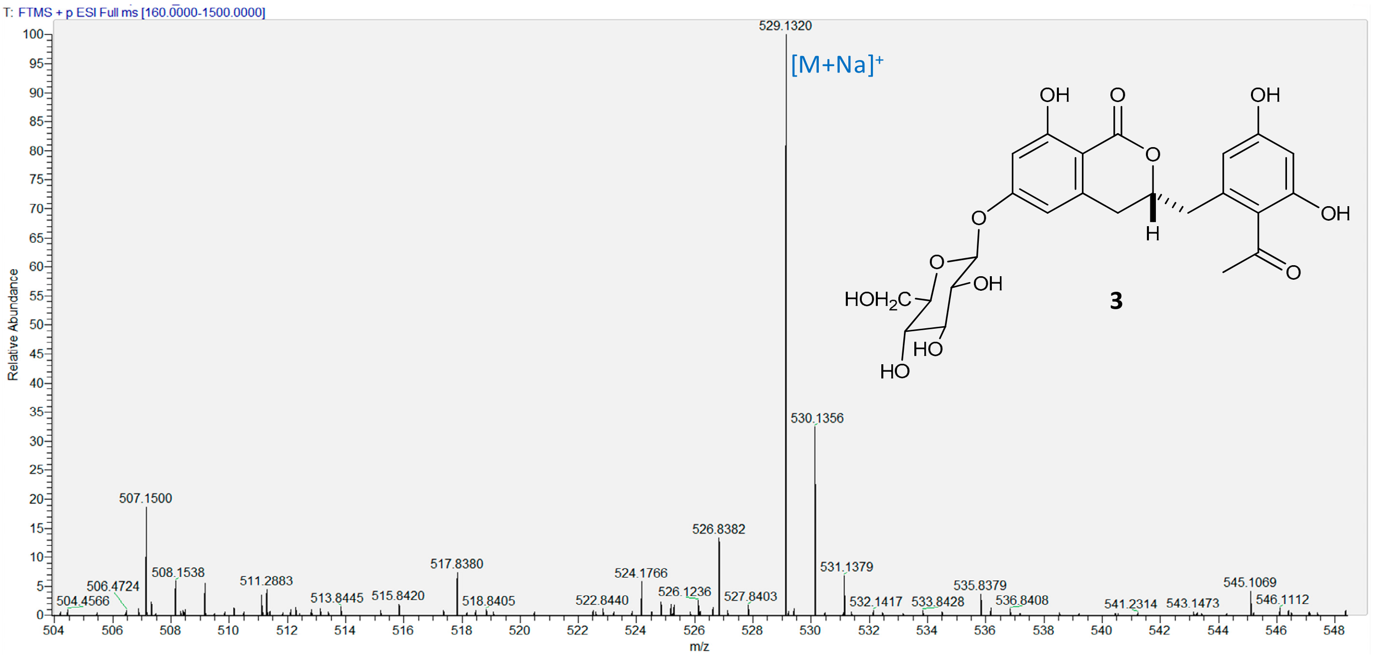

A

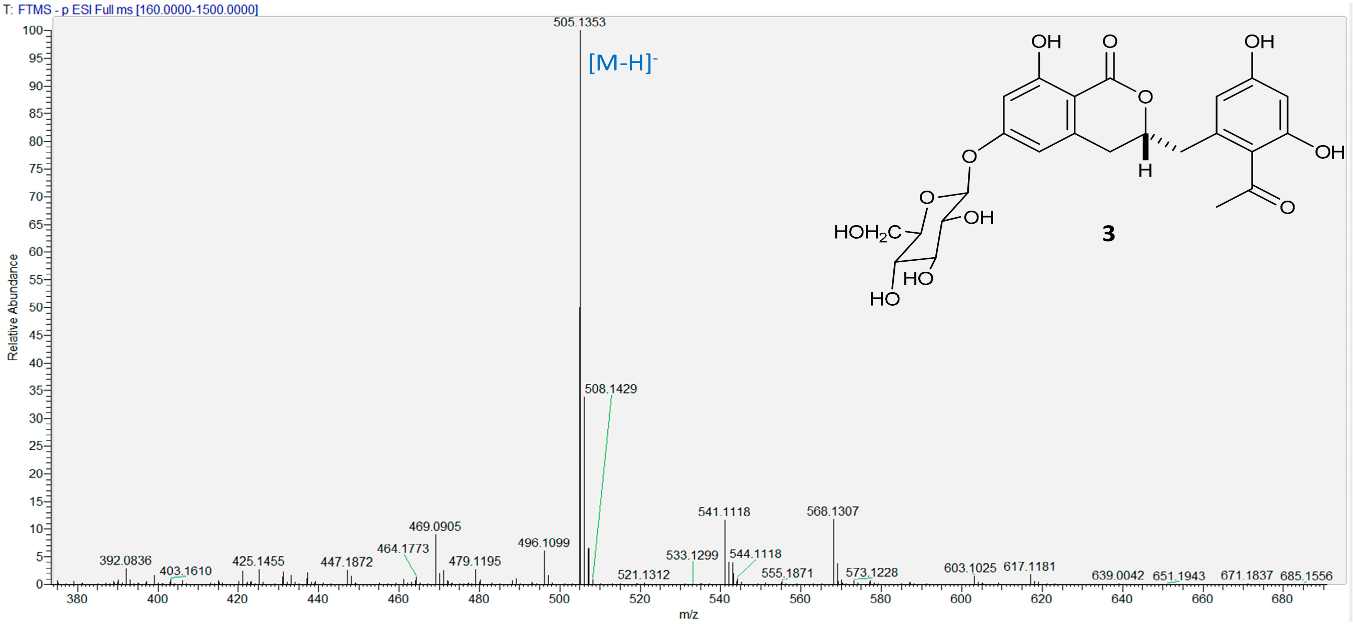

B

Figure 9S: HRESIMS spectrum of compound (**3**) (**A**) positive mode, (**B**) negative mode.

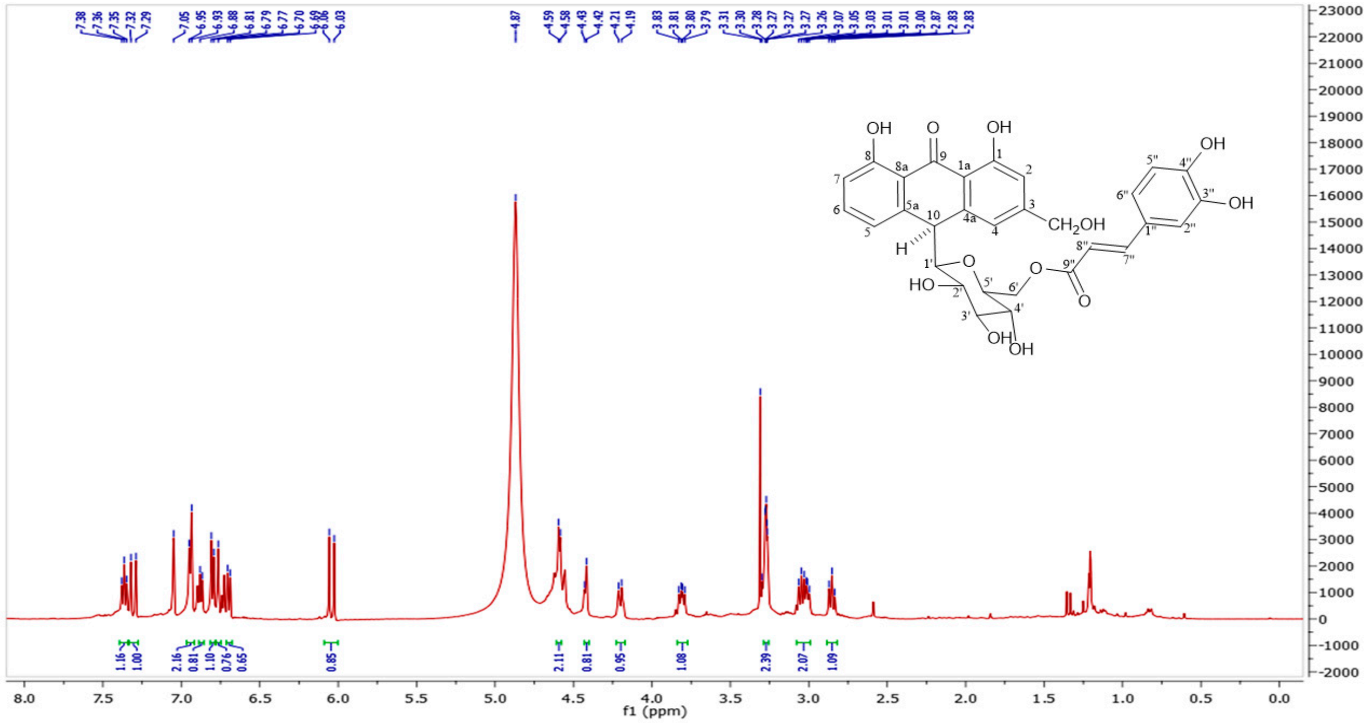

Figure 10S. ^{1}H NMR spectrum of compound (**10**) (500 MHz, CD_3OD)

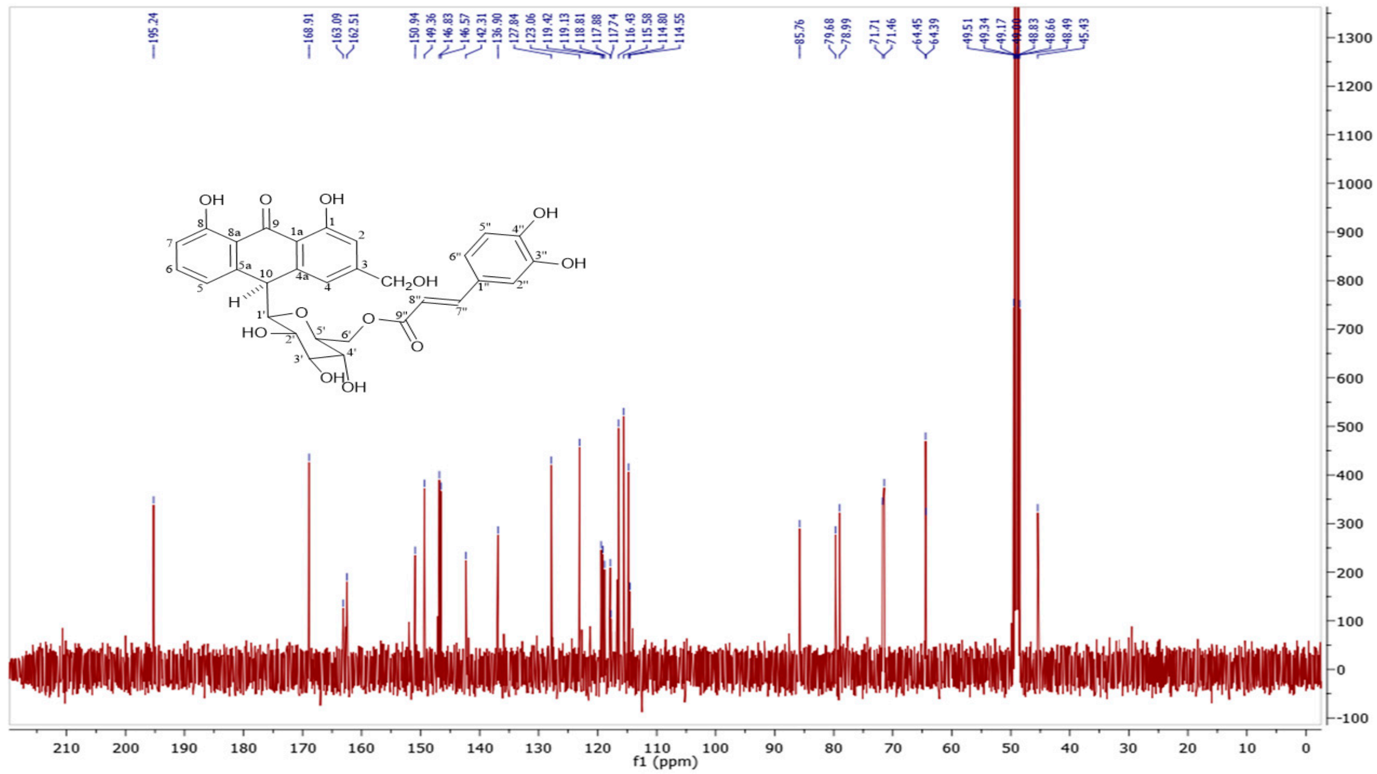

Figure 11S. ^{13}C NMR spectrum of compound (**10**) (125 MHz, CD_3OD)

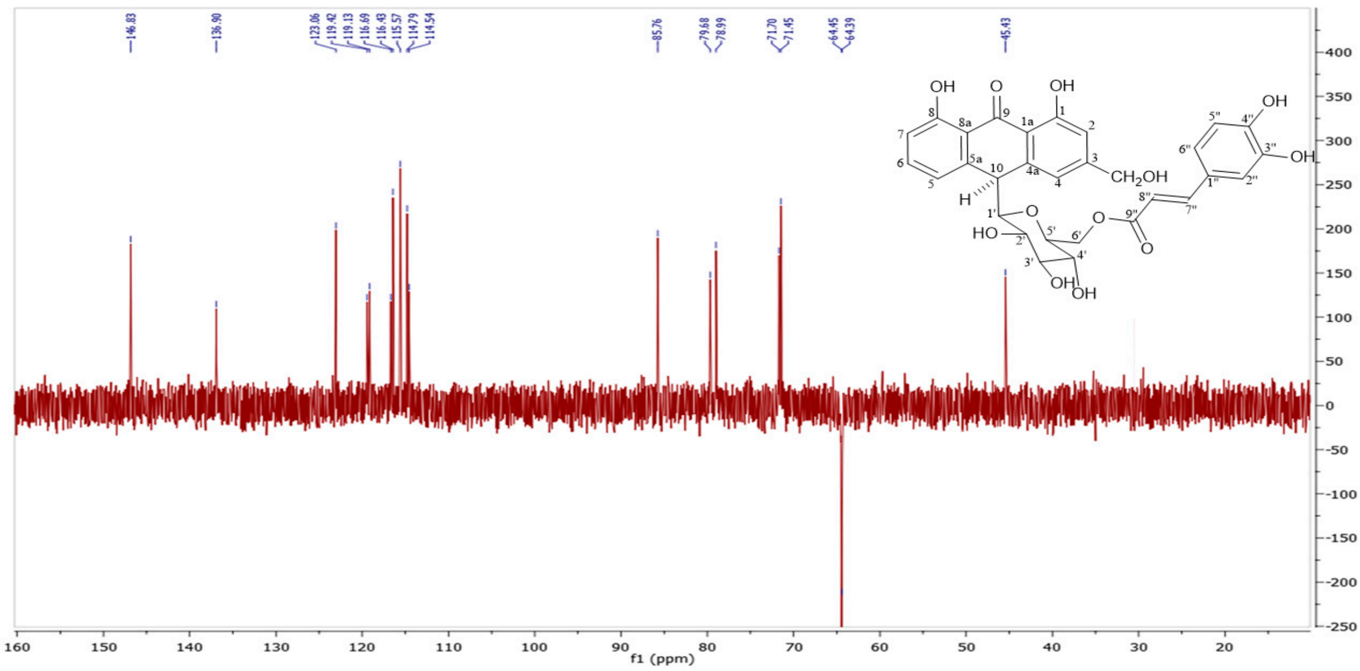

Figure 12S. DEPT ^{13}C NMR spectrum of compound (**10**) (125 MHz, CD_3OD)

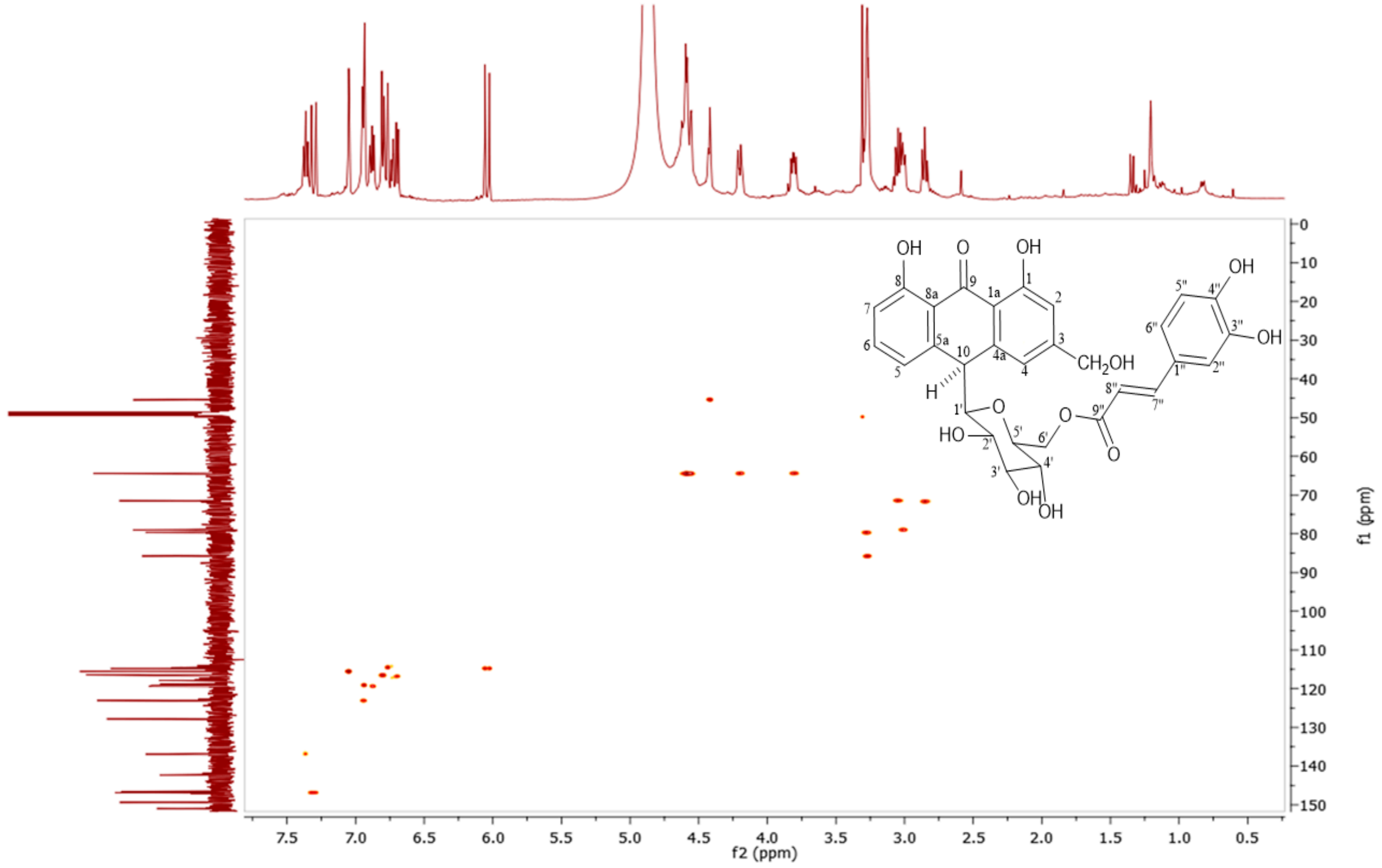

Figure 13S. ^1H-^{13}C HSQC spectrum of compound (**10**) (500 MHz, CD_3OD)

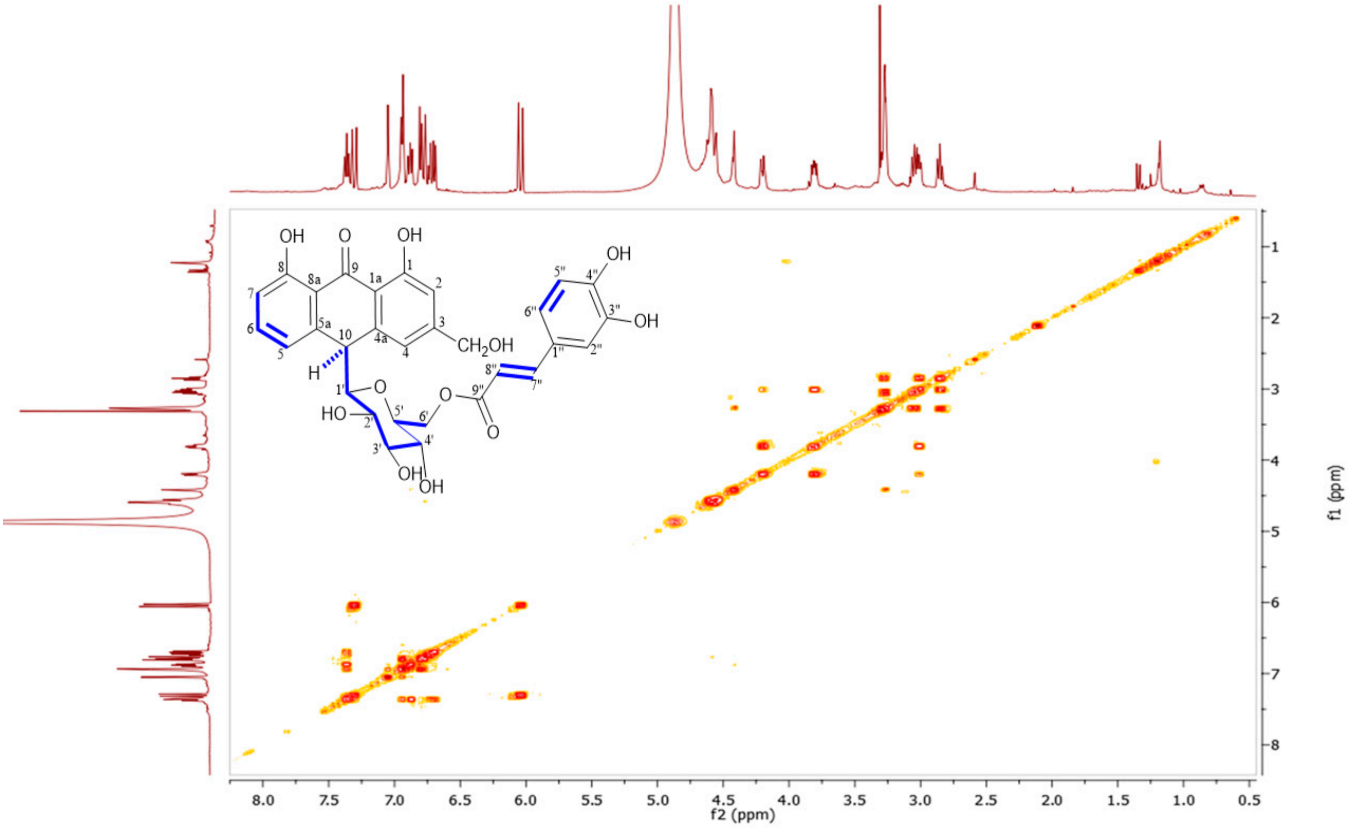

Figure 14S. ^{1}H-^{1}H COSY spectrum of compound (**10**) (500 MHz, CD_3OD)

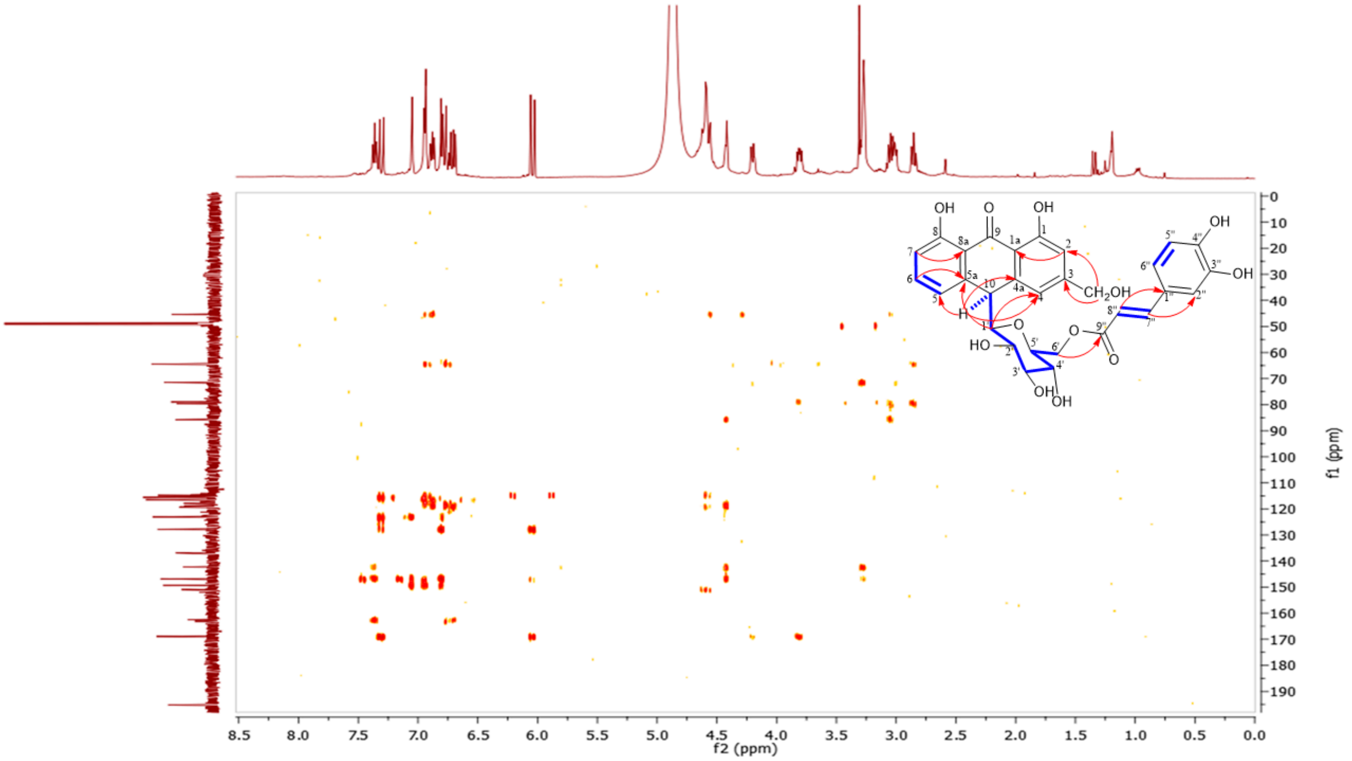

Figure 15S. ^{1}H-^{13}C HMBC spectrum of compound (**10**) (500 MHz, CD_3OD)

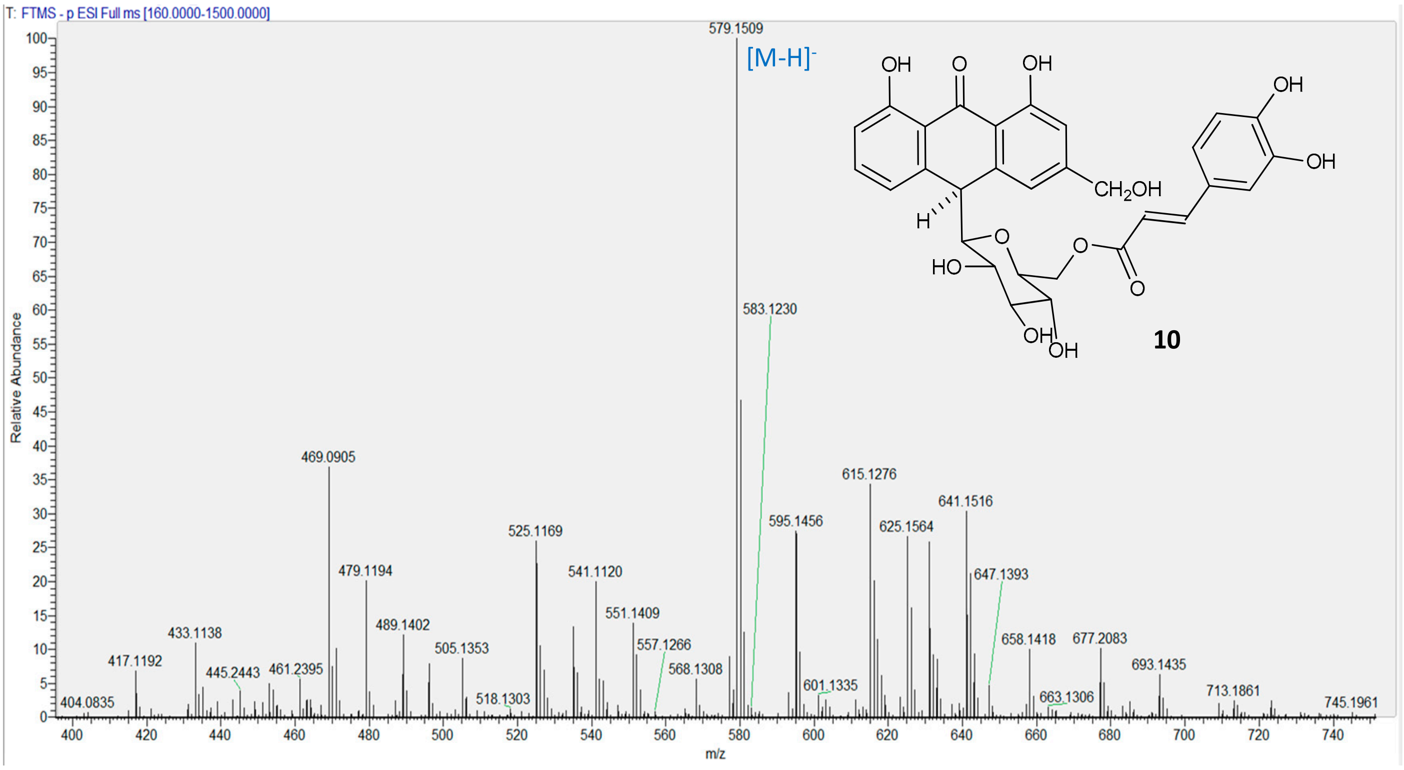

Figure 16S: HRESIMS spectrum of compound (**10**) negative mode.

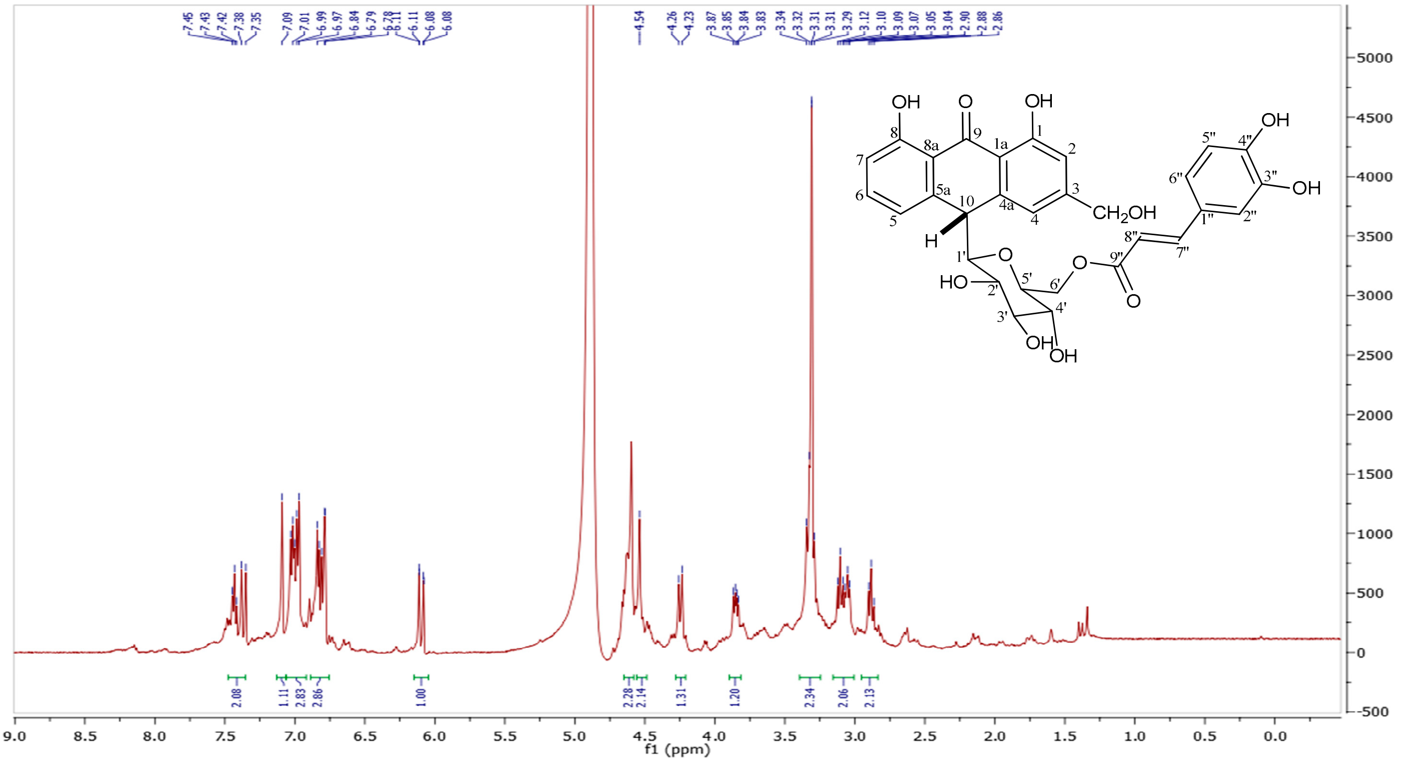

Figure 17S. ^{1}H NMR spectrum of compound (**11**) (500 MHz, CD_3OD)

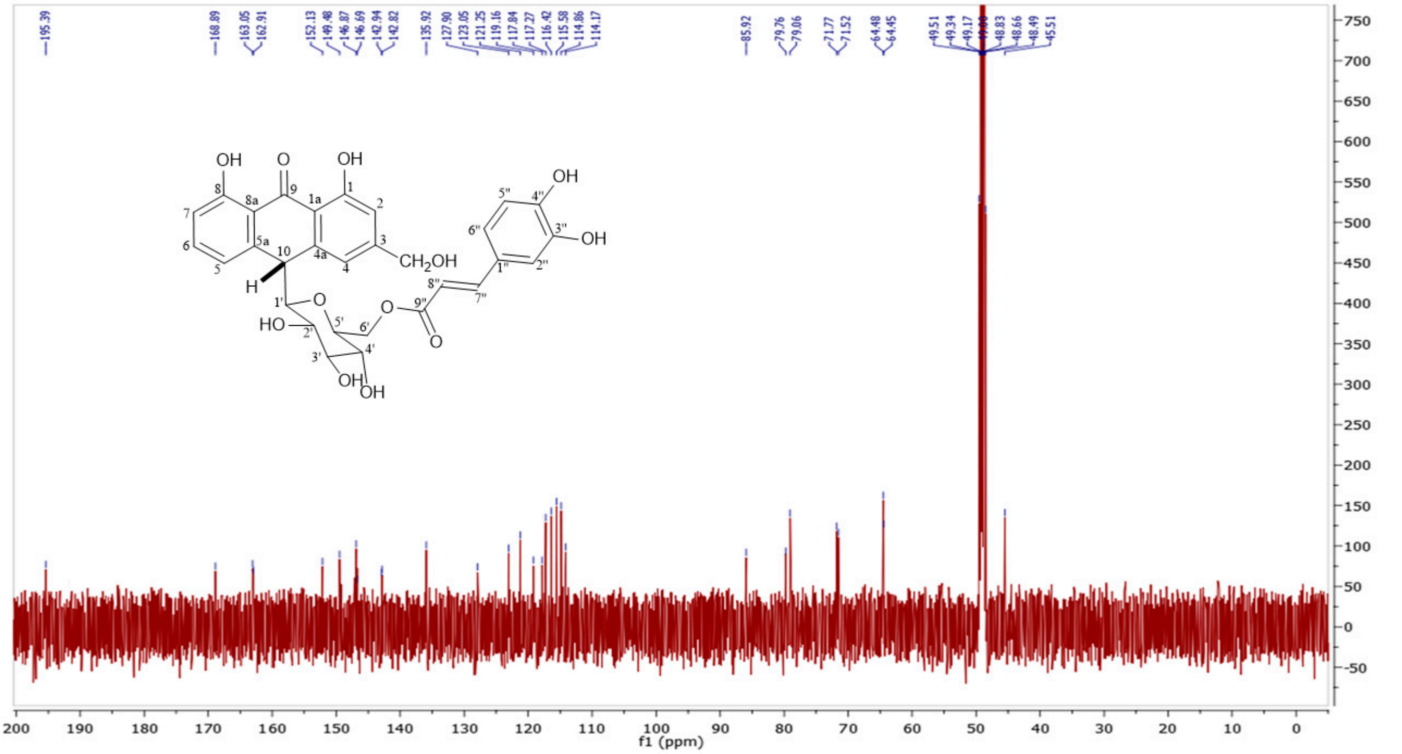

Figure 18S. ^{13}C NMR spectrum of compound (**11**) (125 MHz, CD_3OD)

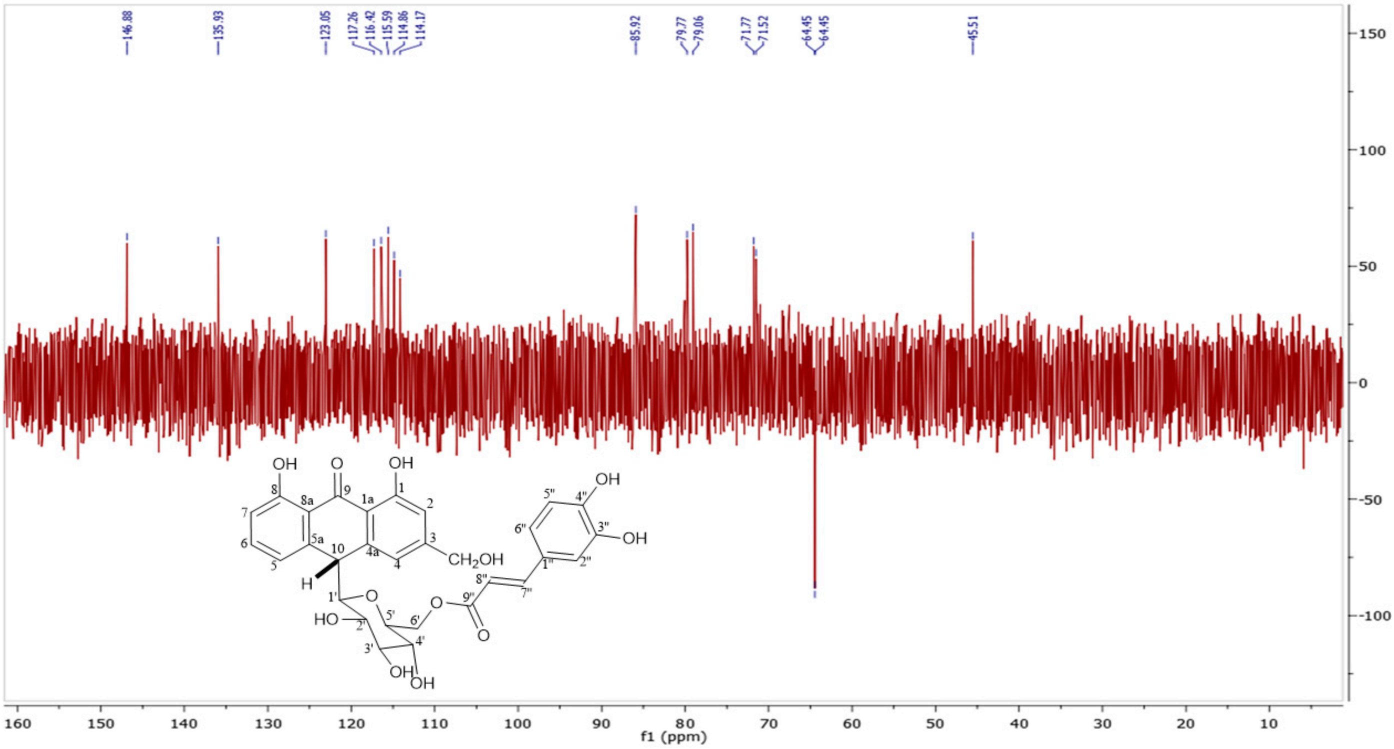

Figure 19S. DEPT ^{13}C NMR spectrum of compound (**11**) (125 MHz, CD_3OD)

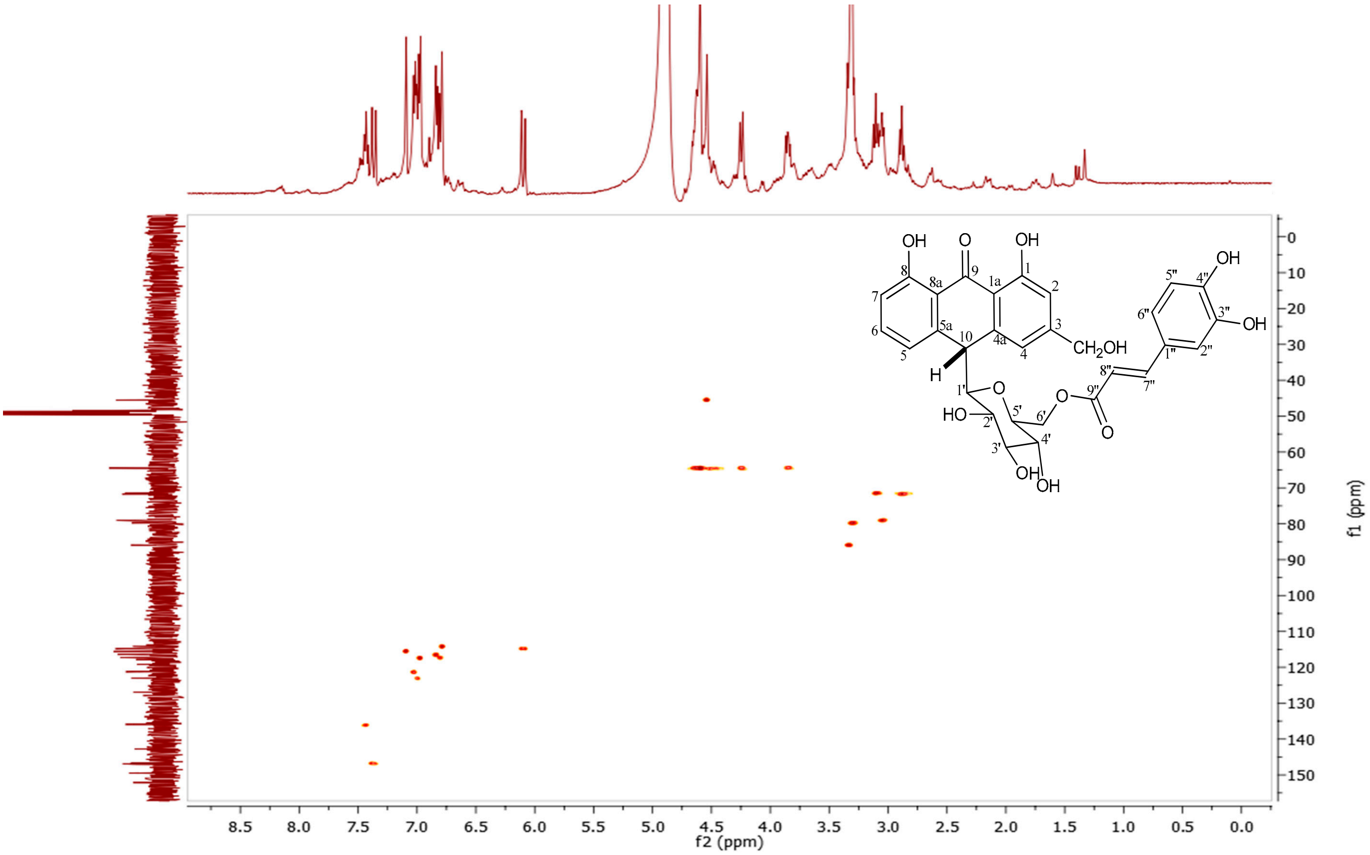

Figure 20S. ^{1}H-^{13}C HSQC spectrum of compound (**11**) (500 MHz, CD_3OD)

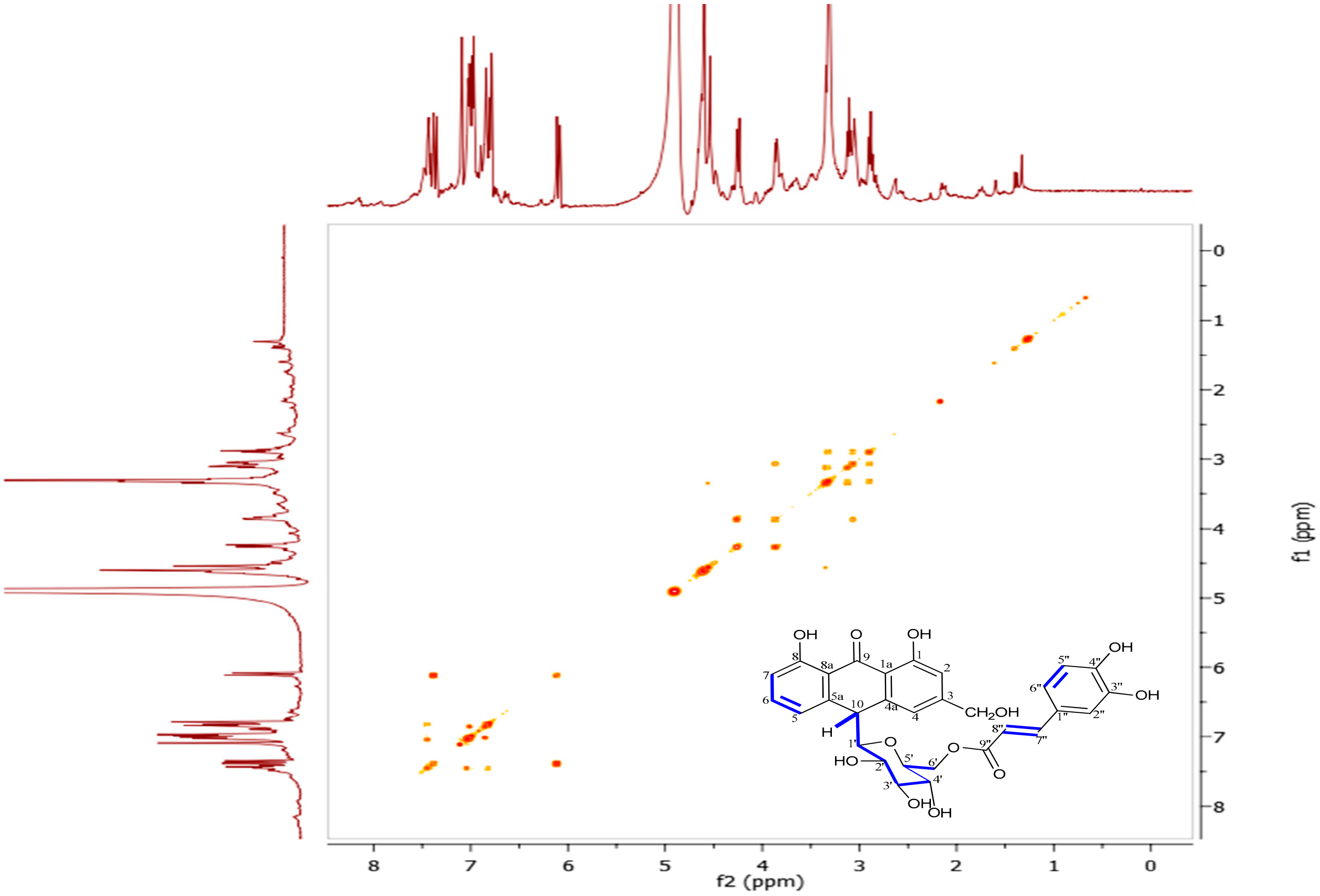

Figure 21S. ^{1}H-^{1}H COSY spectrum of compound (**11**) (500 MHz, CD_3OD)

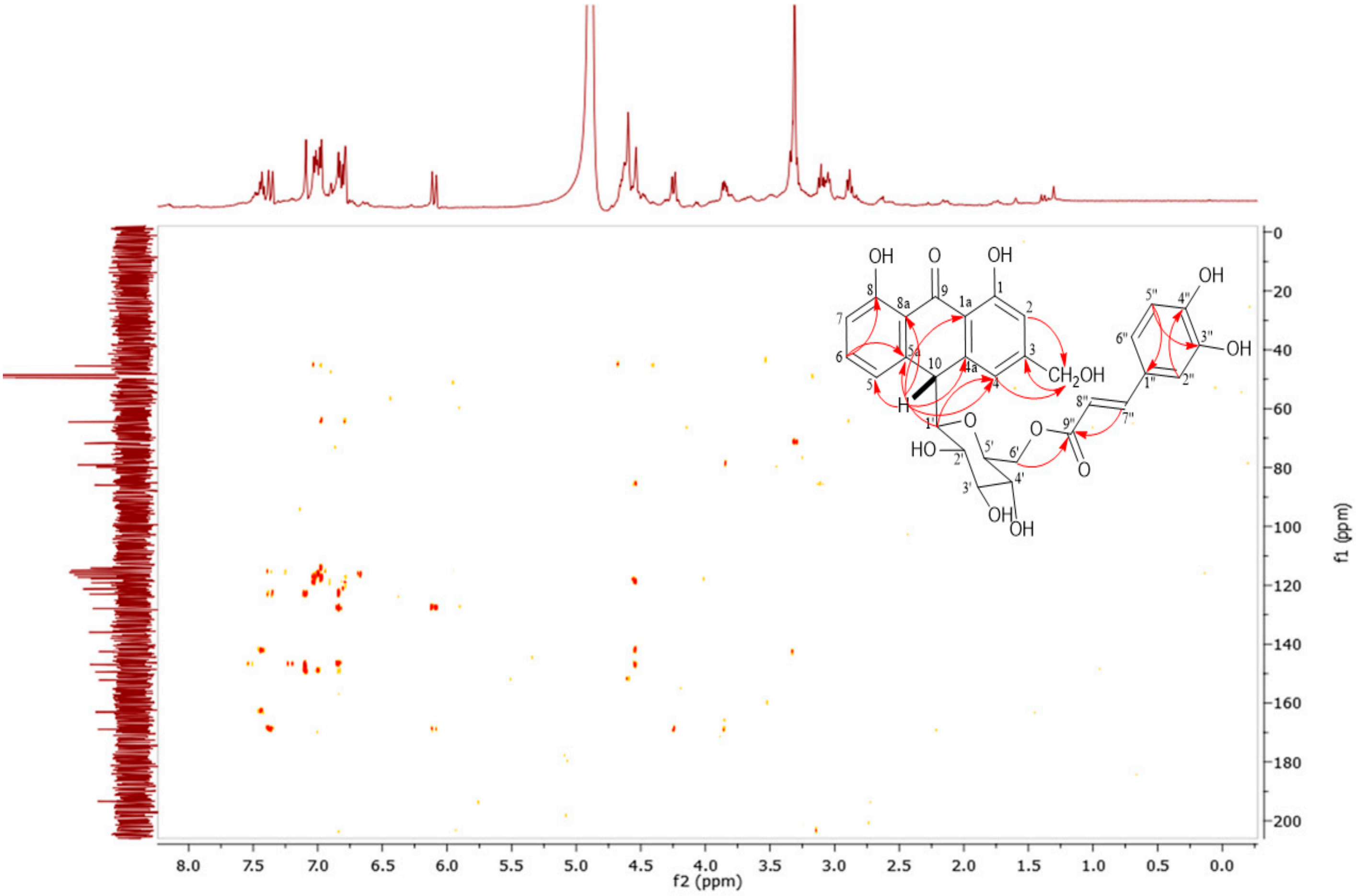

Figure 22S. ^{1}H-^{13}C HMBC spectrum of compound (**11**) (500 MHz, CD_3OD)

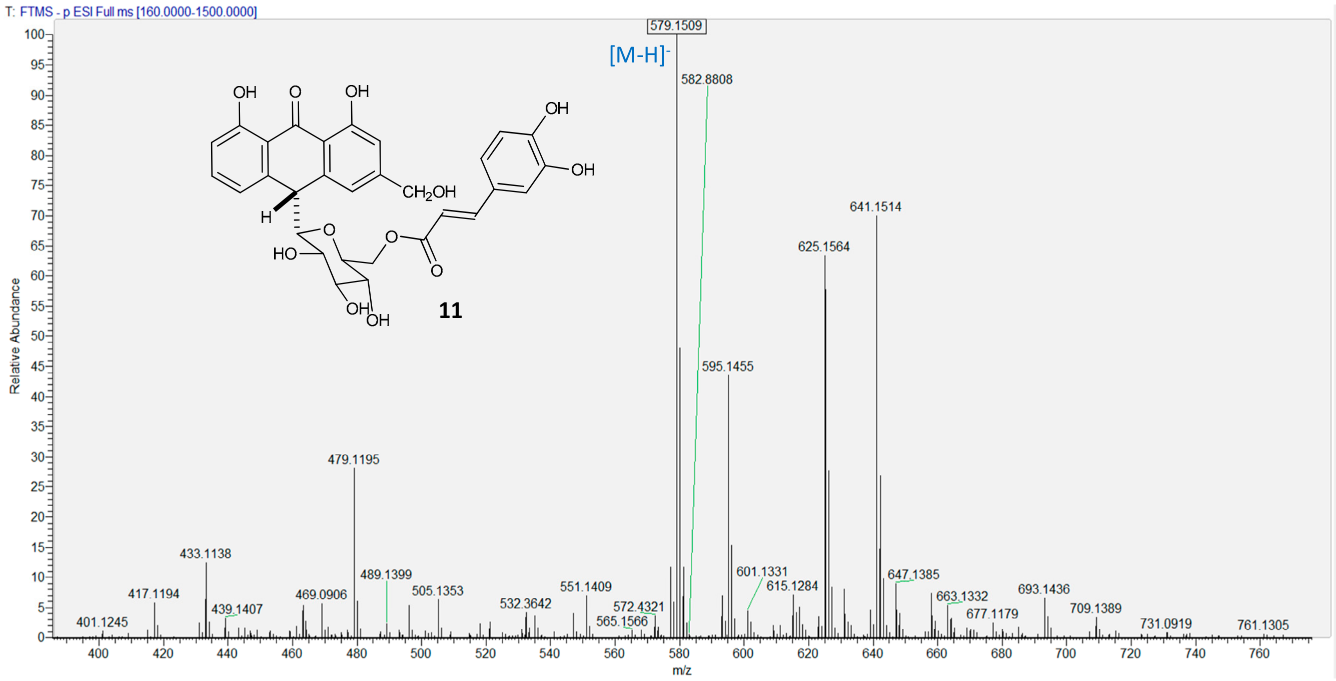

Figure 23S: HRESIMS spectrum of compound (**11**) negative mode

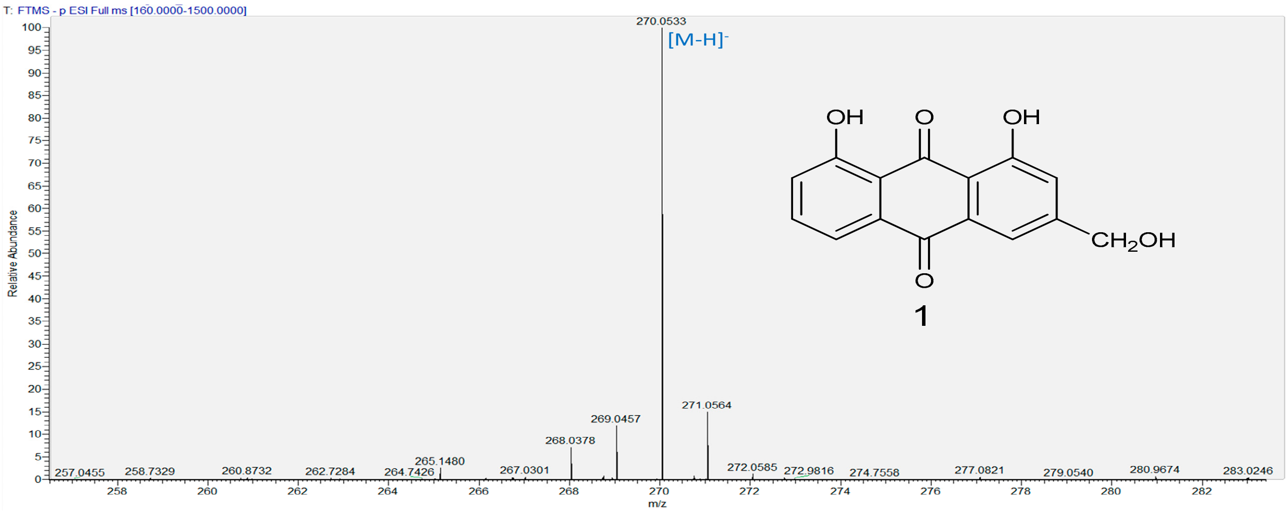
T: FTMS - p ESI Full ms [160.0000-1500.0000]
270.0533
[M-H]-
Relative Abundance
m/z
271.0564
269.0457
268.0378
265.1480
OH
O
OH
CH2OH
1

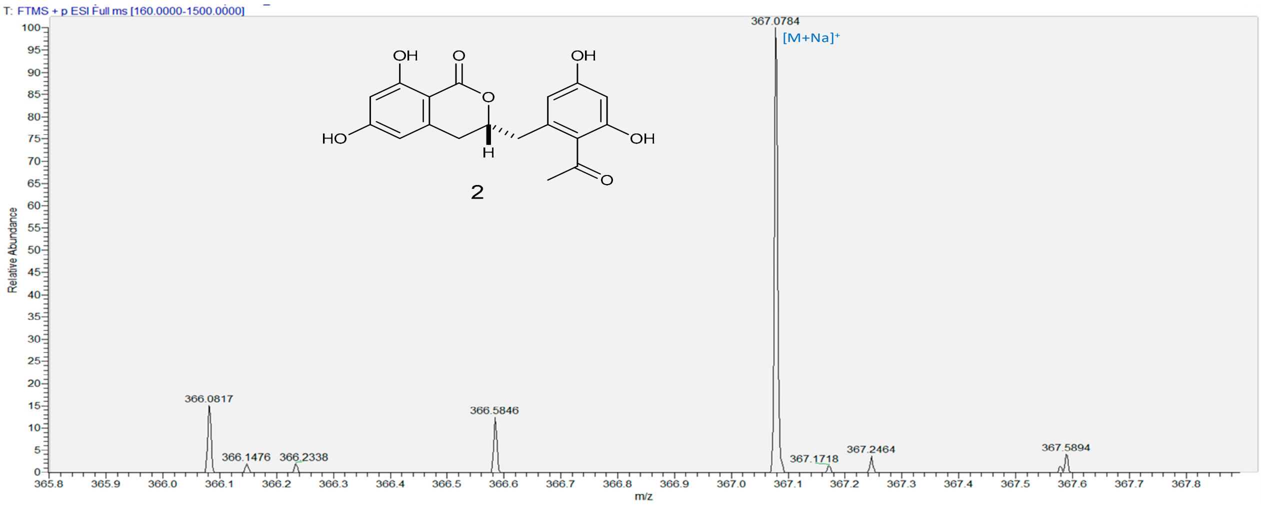
T: FTMS + p ESI Full ms [160.0000-1500.0000]
367.0784
[M+Na]+
Relative Abundance
m/z
366.0817
366.5846
366.1476
366.2338
367.1718
367.2464
367.5894
OH
O
HO
H
OH
OH
O
2

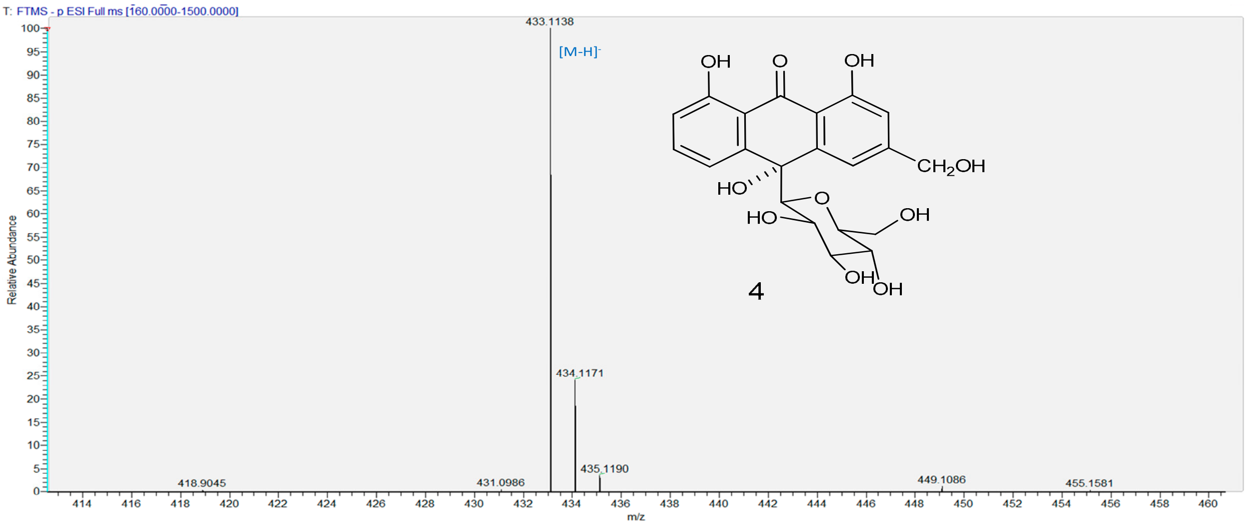
T: FTMS - p ESI Full ms [160.0000-1500.0000]
433.1138
[M-H]-
Relative Abundance
m/z
434.1171
435.1190
418.9045
431.0986
449.1086
455.1581
OH
O
OH
CH2OH
HO
HO
O
OH
OH
OH
4

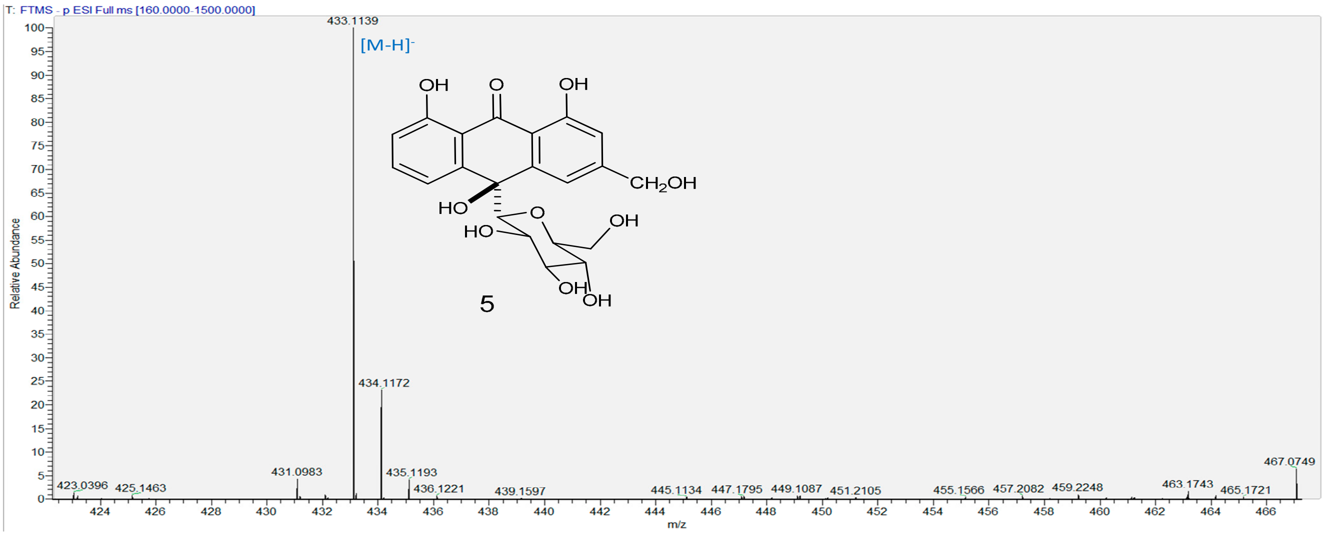
T: FTMS - p ESI Full ms [160.0000-1500.0000]
433.1139
[M-H]-
OH
O
OH
CH2OH
HO
O
HO
OH
OH
OH
5
434.1172
431.0983
435.1193
436.1221
423.0396
425.1463
439.1597
445.1134
447.1795
449.1087
451.2105
455.1566
457.2082
459.2248
463.1743
465.1721
467.0749
Relative Abundance
m/z

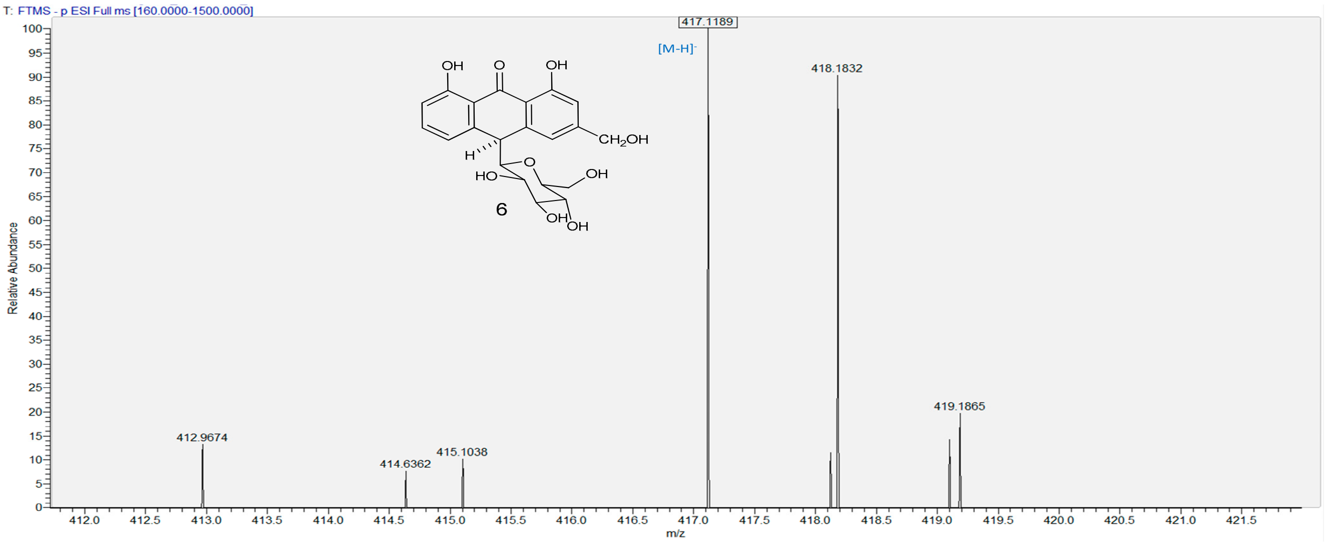
T: FTMS - p ESI Full ms [160.0000-1500.0000]
417.1189
[M-H]-
418.1832
OH
O
OH
CH2OH
H
O
HO
OH
OH
OH
6
419.1865
412.9674
414.6362
415.1038
Relative Abundance
m/z

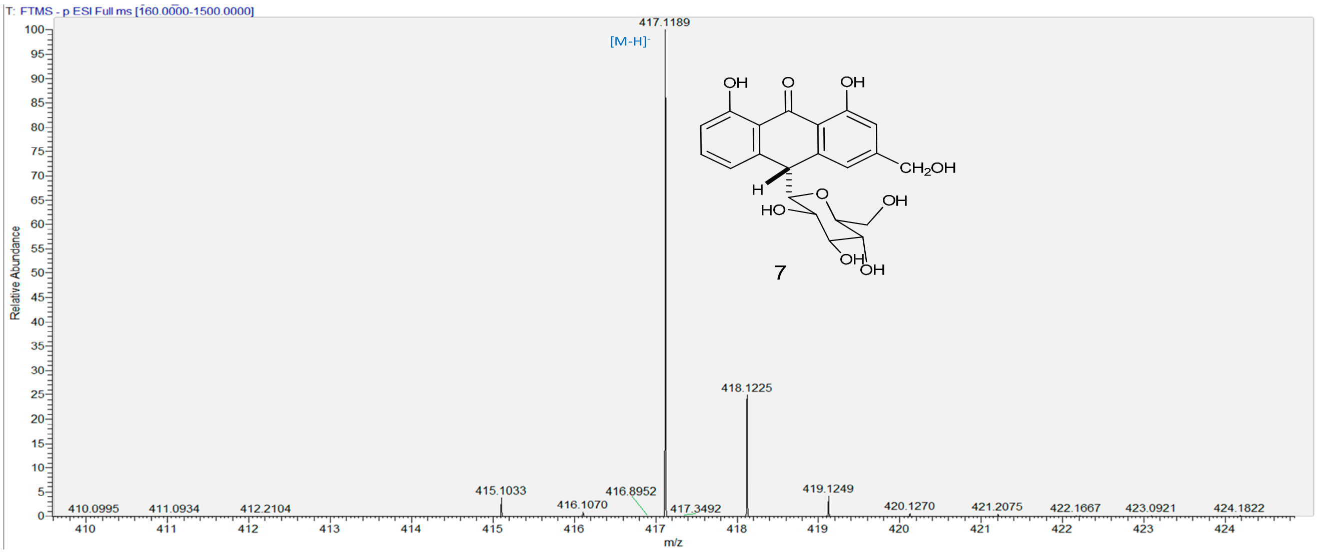
T: FTMS - p ESI Full ms [160.0000-1500.0000]
417.1189
[M-H]-
OH
O
OH
CH2OH
H
O
HO
OH
OH
OH
7
418.1225
415.1033
416.8952
416.1070
417.3492
419.1249
410.0995
411.0934
412.2104
420.1270
421.2075
422.1667
423.0921
424.1822
Relative Abundance
m/z

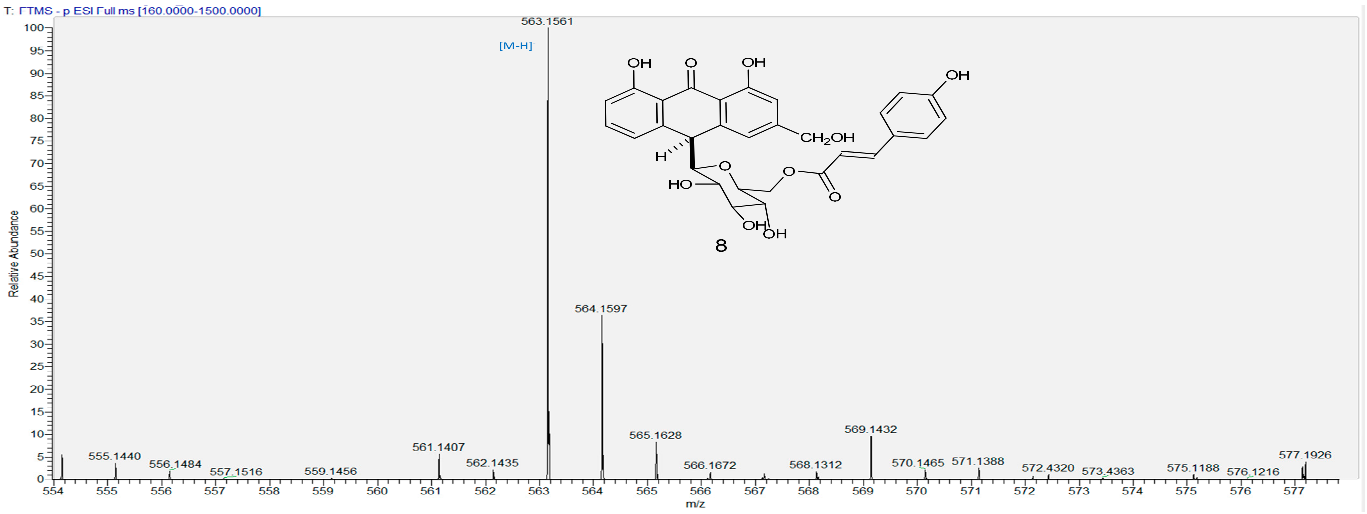

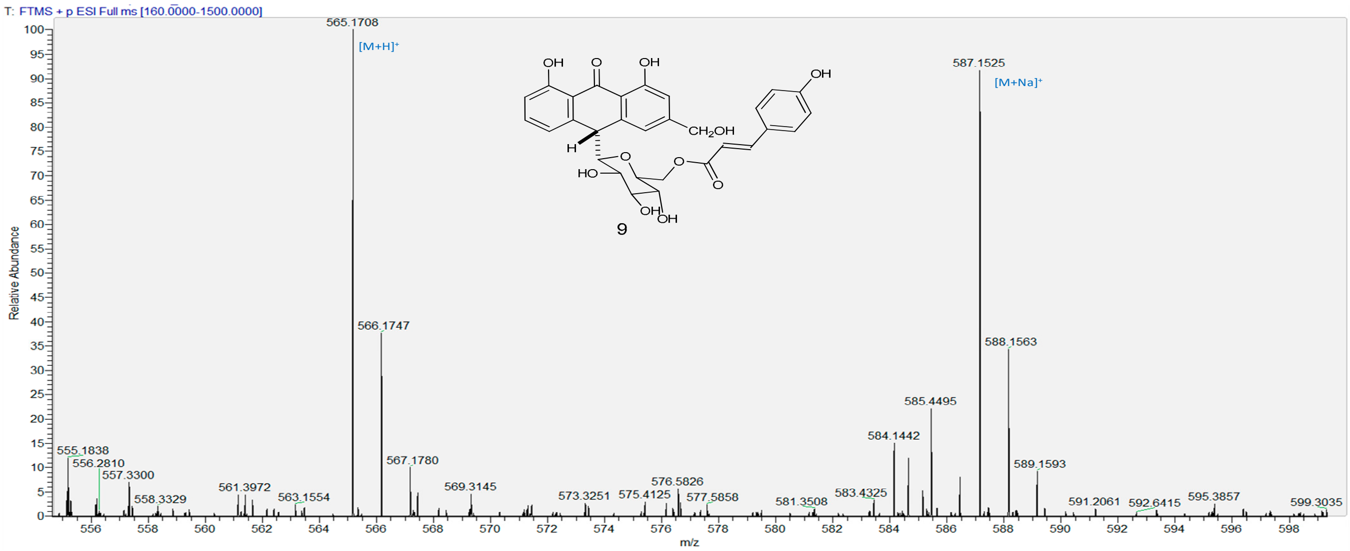

Figure 24S: HRESIMS spectrum of isolated compounds

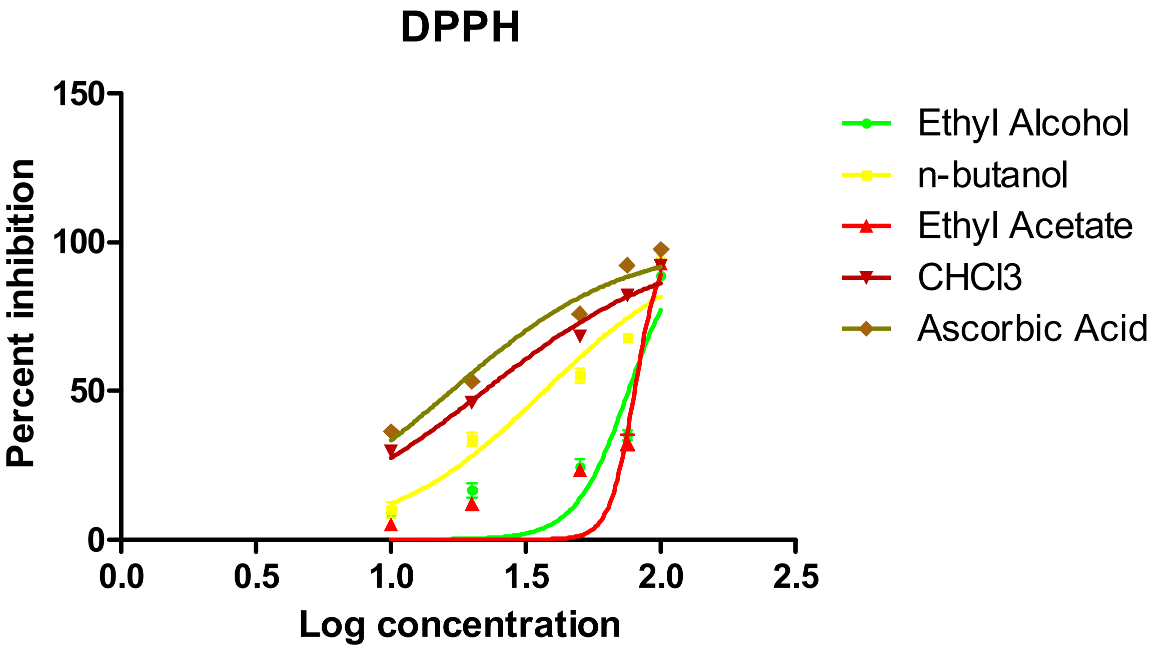

Figure 25S. Scavenging activity of the organic extracts of *A. vacillans* and ascorbic acid using DPPH assay

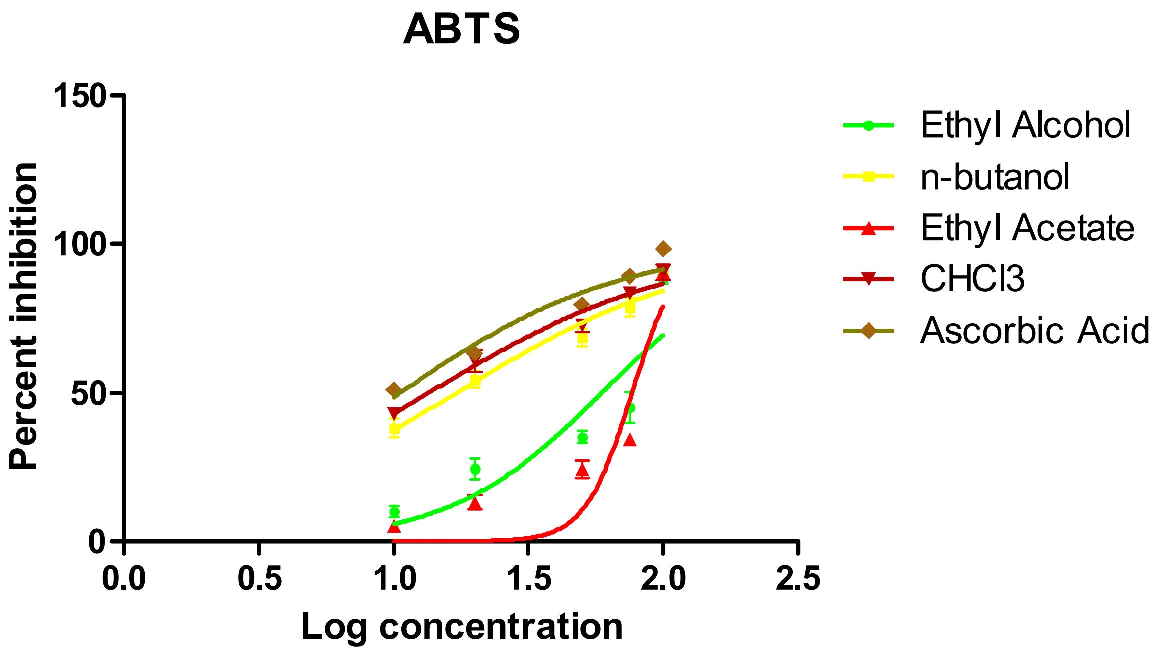

Figure 26S. Scavenging activity of the organic extracts of *A. vacillans* and ascorbic acid using ABTS assay

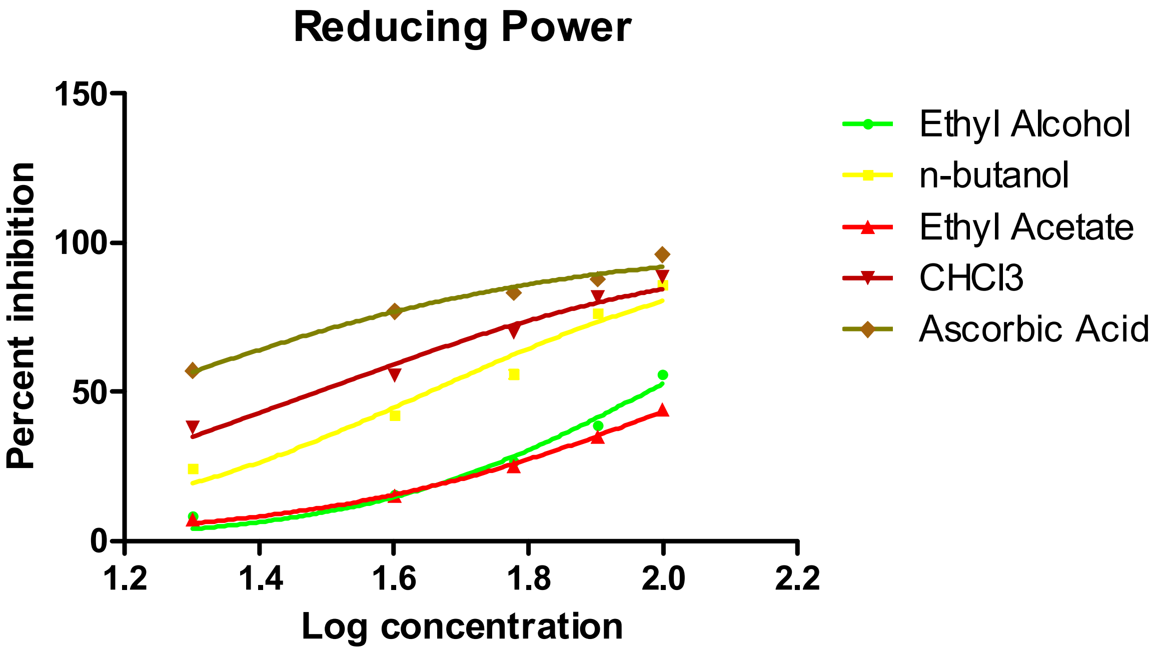

Figure 27S. Reducing power of the organic extracts of *A. vacillans* and ascorbic acid using FRAP method

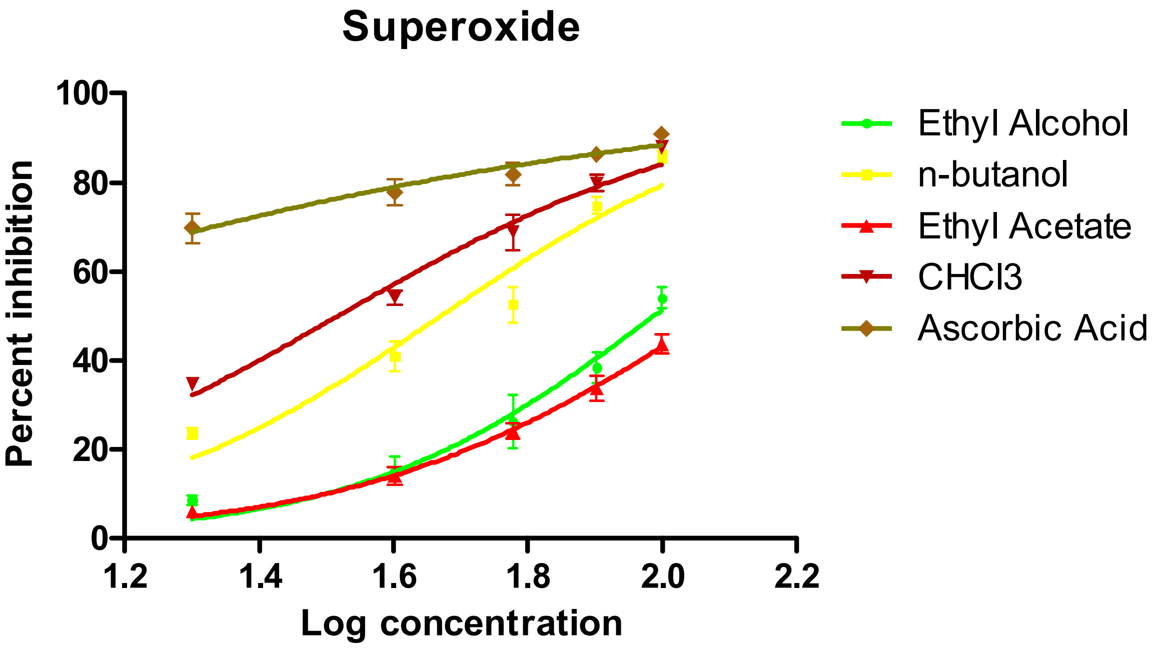

Figure 28S. Scavenging activity of the different extracts of *A. vacillans* and ascorbic acid using superoxide assay

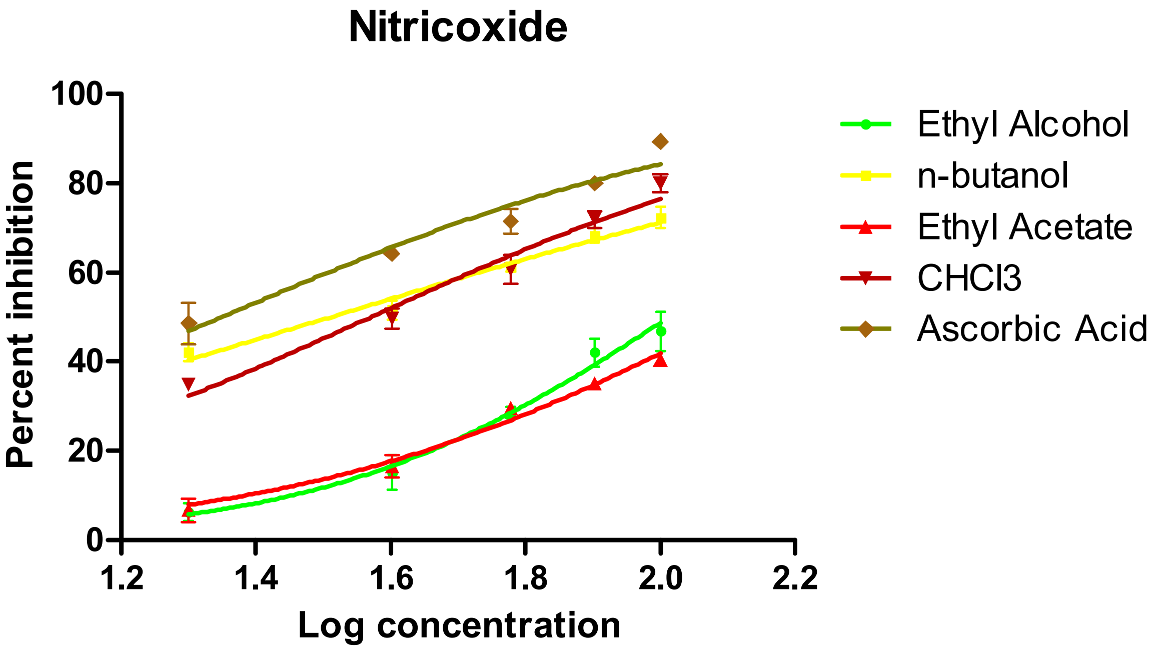

Figure 29S. Scavenging activity of the organic extracts of *A. vacillans* and ascorbic acid using nitric oxide method
